# Effects of fitness qigong and tai chi on middle-aged and elderly patients with type 2 diabetes mellitus

**DOI:** 10.1371/journal.pone.0243989

**Published:** 2020-12-17

**Authors:** Xiaoyuan Li, Hongyu Si, Yamin Chen, Shouhao Li, Ningning Yin, Zhenlong Wang

**Affiliations:** 1 School of Electrical Engineering, Zhengzhou University, Zhengzhou, China; 2 College of Physical Education, Zhengzhou University, Zhengzhou, China; 3 Fitness Qigong Scientific Research Centre of China, Zhengzhou, China; 4 School of Life Sciences, Zhengzhou University, Zhengzhou, China; Universidad Miguel Hernandez de Elche, SPAIN

## Abstract

Currently, qigong and tai chi exercises are the two most common preventive as well as therapeutic interventions for chronic metabolic diseases such as type 2 diabetes mellitus (T2DM). However, the quantitative evaluation of these interventions is limited. This study aimed to evaluate the therapeutic efficacy of qigong and tai chi intervention in middle-aged and older adults with T2DM. The study included 103 eligible participants, who were randomized to participate for 12 weeks, in one of the following intervention groups for the treatment of T2DM: fitness qigong, tai chi, and control group. Three biochemical measures, including fasting plasma glucose (FPG), glycated hemoglobin (HbA1C), and C-peptide (C-P) levels, assessed at baseline and 12 weeks, served as the primary outcome measures. During the training process, 16 of the 103 participants dropped out. After the 12-week intervention, there were significant influences on HbA1C (*F*_2,83_ = 4.88, *p* = 0.010) and C-P levels (*F*_2,83_ = 3.64, *p* = 0.031). Moreover, significant reduction in C-P levels was observed after 12-week tai chi practice (*p* = 0.004). Furthermore, there was a significant negative correlation between the duration of T2DM and the relative changes in FPG levels after qigong intervention, and the relative changes in HbA1C levels were positively correlated with waist-to-height ratio after tai chi practice. Our study suggests that targeted qigong exercise might have a better interventional effect on patients with a longer duration of T2DM, while tai chi might be risky for people with central obesity.

**Trial registration:** This trial was registered in Chinese Clinical Trial Registry. The registration number is ChiCTR180020069. The public title is “Health-care qigong · study for the prescription of chronic diabetes intervention.”

## Introduction

Diabetes mellitus (DM) is a group of metabolic disorders characterized by high blood sugar levels over a prolonged period. Its common and complex clinical syndrome include frequent urination, increased thirst, and increased hunger [1]. If left untreated, diabetes can cause several complications. Acute complications include diabetic ketoacidosis, hyperosmolar hyperglycemic state, or death, and serious long-term complications include cardiovascular disease, stroke, chronic kidney disease, foot ulcers, and damage to the eyes [2].

Among the three main types of DM, type 2 DM (T2DM) is the most common, accounting for approximately 90% of cases. The rates of T2DM have increased markedly since 1960 in parallel with obesity [3]. According to the International Diabetes Federation, approximately 392 million people worldwide have diabetes, and more than 629 million people are projected to have diabetes by 2045 [4]. Typically, diabetes begins with insulin resistance and usually occurs at a middle or older age, and as the disease progresses, it may lead to insulin deficiency [5–7]. Several studies have indicated that T2DM primarily occurs because of obesity and lack of exercise and is associated with a 10-year-shorter life expectancy [8–11]. Evidence-based guidelines suggest that DM is typically managed with multidisciplinary therapies, involving medication, education, psychological and emotional therapy, and exercise [12–14].

Although medication is beneficial for T2DM and has been advocated as a core component for diabetes treatment [15–17], some patients continue to inject and administer oral insulin when blood sugar levels are not adequately controlled [18], leading to low blood sugar and serious complications [19]. Hence, new exercise therapies are needed for controlling blood sugar levels and for improving their physical and emotional functioning and quality of life.

The American Diabetes Association (ADA) report indicates that physical activity is beneficial for patients with T2DM [20]. Tai chi has been practiced for nearly 600 years in China; it incorporates slow dance-like movements and integrates musculoskeletal, breathing, and meditation training [21]. Because of its mind-body integration, tai chi seems to be appropriate for the treatment of chronic T2DM [22]. Recently, some research have shown that regular tai chi exercise for a few months can significantly decrease the levels of fasting plasma glucose (FPG) [23] and glycated hemoglobin (HbA1C) [24], albeit not clinically normalized. Simultaneously, tai chi can provide an even better improvement in both metabolism and immunity in T2DM patients [25]. However, other studies have indicated that tai chi exercise did not significantly reduce inflammatory biomarkers in diabetic patients [26]. Further studies for identifying another moderate exercise with improved treatment effects for T2DM patients are needed.

Qigong is an important part of traditional Chinese medicine with a thousand-year history and can be considered an ancient practice of mind-body integration and refinement of one’s vital energy or life force for optimal health and personal development [27]. Compared with the complicated movements and power actions of tai chi, qigong is more suitable for older people with its soothing Qi circulation and meditation. Relevant studies have suggested the favorable effects of qigong on blood glucose, HbA1C, 2-hour plasma glucose, insulin sensitivity, and blood viscosity [28, 29].

This single-blinded, randomized, controlled trial was conducted for analyzing the physical benefits of qigong and tai chi in middle-aged or older patients with T2DM. The primary objective was to evaluate the efficacy of 12-week tai chi and qigong interventions on three biochemical measures in adults aged 40 years or older; the major physiological states of patients affecting treatment outcomes were analyzed as well.

## Methods

### Study participants

Participants were recruited from a pool of diabetes patients enrolled in a tertiary-care academic hospital in China between November 2016 and October 2017. Eligible patients were 40 years old or older and fulfilled the new criteria for the diagnosis and classification of diabetes, as revised by the ADA and the World Health Organization (WHO), and were formally adopted by the Chinese Diabetes Association in 1999 [30–32]. These criteria included a history of T2DM with a minimum three-month duration, inactive (defined as not being involved in any moderate or strenuous activity in the previous three months) [33], can independently ambulate, and no cognitive impairments [34]. Patients who participated in tai chi and qigong training within the past six months and those with serious medical conditions that might limit their participation were excluded. Participants were allowed to continue routine medications and maintain usual visits to their fitness physicians throughout the study.

### Study design

The members of our research team made an initial telephone contact and screened potential participants for determining patient’s interest and eligibility for the study. Researchers who contacted potential participants for informed consent were not involved in other research processes (such as random assignment, provision of intervention, and baseline assessment). The study was a single-blinded, randomized, controlled trial involving three intervention groups: fitness qigong, tai chi, and stretching control. Patients who agreed to participate in the study were randomized to one of the three groups using computer-generated numbers. The randomized treatment assignments were concealed from all participants until they were scheduled to undergo baseline assessments. Each group included a 60-minute practice five times per week for 12 consecutive weeks. The intervention phase lasted 12 weeks between November 2017 and January 2018, in which exercise classes were provided. The intervention was primarily designed for investigating the efficacy of qigong, tai chi, and low-level stretching activity for decreasing and controlling hyperglycemia.

**Fig 1:**
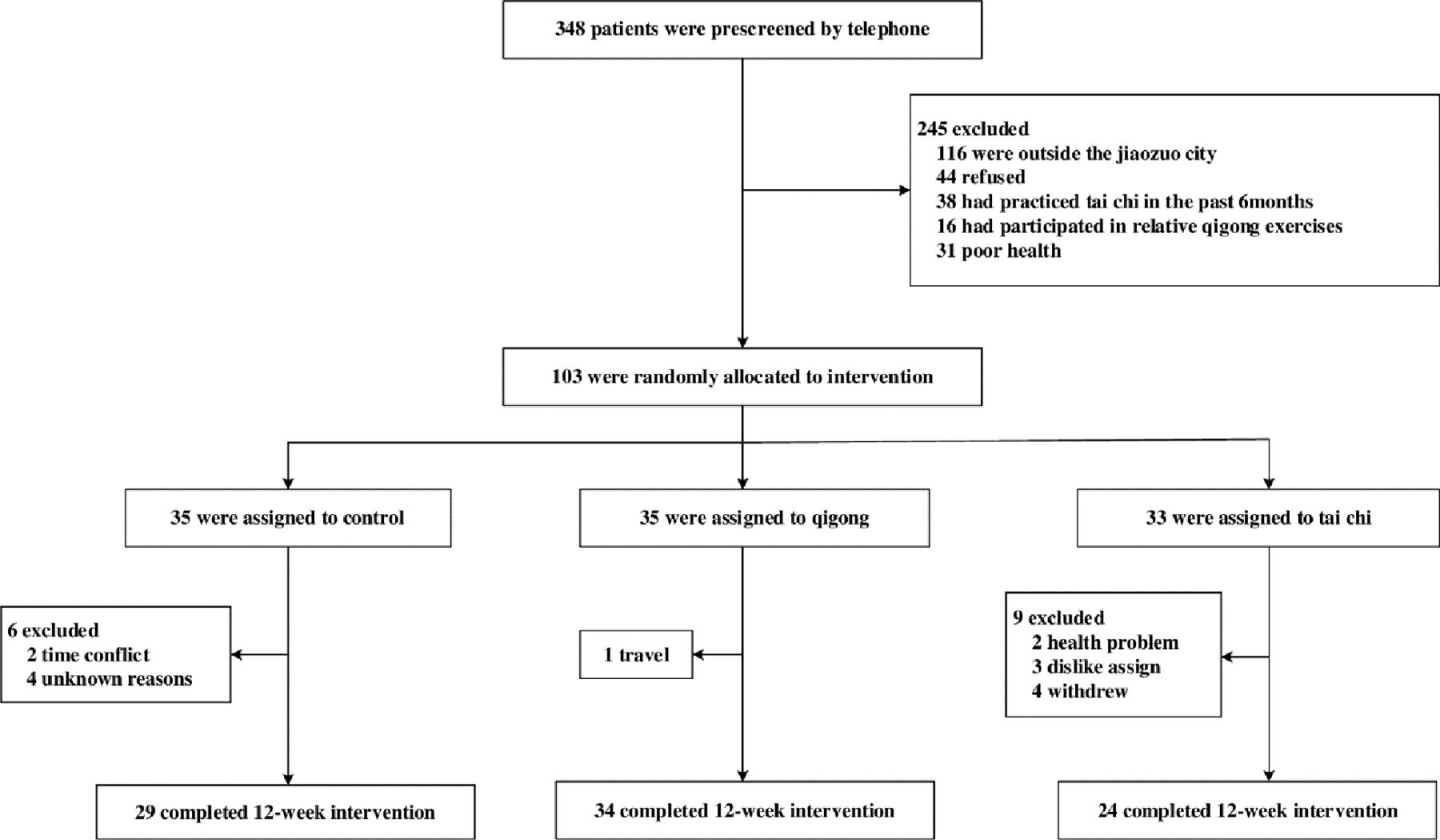
Screening, randomization, and completion of the 12-week intervention.

The assessors of this study who oversaw the collection, management, and analysis of the data were blinded to the design and conduct of the study; they were also not involved in randomization or intervention training. All assessments and surveys were made at a prescheduled appointment with each participant. The institutional review committee of Zhengzhou University approved the research protocol, and all study participants provided informed consent before they were enrolled in the study. The study methods and procedures were conducted in accordance with the trial protocol. However, there was a slight deviation in the number of participants after enrollment. Owing to time conflicts, health problems, dislike of assignment, etc., the number of participants in each group was slightly reduced. Finally, 29 patients in the control group, 34 in the Qigong group, and 24 in the tai chi group completed the trial. The study flow and retention of study participants throughout the 12-week intervention trial is summarized in Fig 1. From May 2017 to July 2017, the preparation and approval of the project and staff training were initiated. From August to September, a telephone interview was conducted from the case bank of the hospital for completing the investigation and consultation of the participants. In October 2017, the screening was completed, unqualified participants were excluded. The qualifying subjects who agreed to participate in the study were randomly assigned to one of the three groups using computer-generated numbers. Within the next 2 weeks, they were then notified to sign an informed consent. From November 2017 to January 2018, a three-month training intervention officially began. The follow-up time was once a week from November 2017 to January 2018.

### Intervention

Fitness Qigong: The fitness qigong exercise program in the study was conducted and choreographed by the qigong research team, jointly composed of the College of Physical Education of Zhengzhou University and fitness Qigong Scientific Research Center of China, based on the classical Qigong maps on physical and breathing exercises in ancient China, such as guided maps [35], Ling Jian Zi [36] and Huashan Rock Art [37]. Compared with tai chi, qigong exercise was designed based on the theory of meridian in traditional Chinese medicine, aiming at the characteristics of diabetic patients. Each movement of qigong is based on the regularity of meridians' circulation, which highlights the characteristics of dredging different meridians for preventing and treating various diseases of internal organs. The fitness qigong classes were taught by a member of the Fitness Qigong Association of Jiaozuo, which have professional teachers with more than 10 years of qigong teaching experience. Before introducing the formal practice of the exercise, the teacher first taught the theory of qigong and its movement essentials. The participants were then provided with printed materials on the principles and techniques of qigong. In the subsequent week, participants practiced the whole form of self-created qigong under the teacher’s instruction. Each session included a warm-up and self-massage for 10 minutes, followed by a review of movement principles and breathing techniques for 10 minutes, practice for 30 minutes, and relaxation for 10 minutes. Throughout the intervention period, participants were instructed to practice qigong at home for about an hour, consisting of warm-up, rest, and 30 minutes of practice each day. The home practice exercise sessions were monitored via mobile phones that were used to clock in. Intensive guided exercises were conducted every weekend to follow up and collect feedback information. At the end of the 12-week intervention, participants were encouraged to maintain their qigong exercise using an instructional DVD.

Tai Chi: For the tai chi intervention, the classical Chen style (18 forms) was selected [38]. The tai chi classes were taught by an experienced instructor with more than 10 years of teaching experience. Each session included a warm-up and self-massage for 10 minutes, followed by a review of principles and movement essentials for 10 minutes, practices and skills for 30 minutes, and relaxation for 10 minutes. The course process and training method were the same as those of the qigong group. All participants were instructed to complete the tai chi exercises for a consecutive 12-week intervention.

Stretching Control: The stretching course, taught by a health professional and qualified exercise instructor, comprised health education for 20 minutes and 40 minutes practice for five times in the first week [39, 40]. A didactic lesson on a topic related to DM included the diagnostic criteria, causes and risks of the disease, disease control, diet and nutrition, physical and mental health, and fitness and lifestyle management [41]. Forty minutes of each class included warm-up and self-massage for 10 minutes and stretching exercises for 30 minutes supervised by the instructors. The low-intensity program consisted predominantly of upper-body, trunk, and lower-body stretches, and controlled breathing and relaxation. The weekly schedule and class format were identical to those of qigong and tai chi for the subsequent 12 weeks.

### Measurement of parameters

Before the initial data collection, all data collectors and phlebotomists from the Second People’s Hospital of Jiaozuo were trained using a common standardized operating protocol. Before participating in different trainings, the basic information and anthropometric parameters of the patients, including age, duration of DM, weight (kg), and height (m) were recorded. The primary outcomes consist of three biochemical measures, including FPG, HbA1C, and C-peptide (C-P) levels [42]. These parameters were measured at baseline and at the end of the 12-week intervention. Patients were instructed to collect blood samples within one week after the end of the 12-week intervention training. For blood sample testing, patients were notified in advance, and the whole process was completed within a week for ensuring the authenticity and reliability of the samples. Plasma glucose levels were analyzed with a chemical analyzer (Beckman Coulter AU680, Beckman Coulter, CA, USA). The HbA1C fraction was measured using an immunofluorescence quantitative analyzer (Getein1100, Getein Biotech Inc., Nanjing, China). C-P levels were measured using an automatic chemiluminescence instrument (MAGLUMI 4000 Plus, Snibe, Shenzhen, China).

Body mass index (BMI) (body mass divided by body length squared) and waist-to-height ratio (WHtR) were calculated for each patient [43, 44]. The relative changes (defined as the ratio of the post-experiment value to the baseline) in the three biochemical parameters were calculated for regression analysis of the biochemical measures for different interventions.

### Statistical analyses

All statistical analyses were performed using the SPSS statistical program, version 19.0. Categorical variables (sex and medication) were expressed as frequencies or proportions. All other anthropometric parameters and biochemical results were presented as mean ± standard deviation. The normality of the data distribution was tested using the Kolmogorov–Smirnov one-sample test. The analysis of covariance (ANCOVA) was used for controlling the possible effect of pretest data. The least-significant difference (LSD) multiple comparisons test was adopted for determining statistical significance among the three intervention groups. The linear regression analysis was used for determining how the patients’ age (years), duration of DM (years), BMI (kg/m^2^), or WHtR influenced the three biochemical measurements (FPG, HbA1C, and C-P) after different interventions. The statistical significance was set at a *p-value* of <0.05.

## Results

Of the 348 patients who met the age and T2DM eligibility, 245 (70%) patients were not included: 116 were living outside the city, 44 refused to participate, 31 were in poor health, 38 practiced tai chi, and 16 practiced qigong exercises in the past six months. One hundred three (30%) eligible patients were randomly assigned to the tai chi group (*n* = 33), qigong group (*n* = 35), and the control group (*n* = 35). During the training process, 16 (15.5%) of the 103 randomized participants dropped out (tai chi group, *n* = 9; qigong group, *n* = 1; stretching control group, *n* = 6) for health reasons, dislike of the assignment, time conflict, travel, and other reasons. No adverse events occurred during the course of the study. The minimum level of attendance was more than 50 sessions (>70% attendance level) for participants. Finally, post-experiment data were merged with baseline data, yielding 87 participants who were examined during both the periods as the study group: control, *n* = 29 (17% dropout rate); tai chi, *n* = 24 (27% dropout rate); and qigong, *n* = 34 (3% dropout rate).

### Baseline characteristics

The baseline data for the 87 participants randomized into three groups who agreed to complete the entire experimental phase are presented in Table 1. The age of the participants ranged 40–77 years (mean age, 59.91 ± 8.36 years), and 54% of the patients were men. The mean BMI was 25.05 ± 2.79 kg/m^2^, and the mean WHtR was 0.53 ± 0.05. The duration of patients with T2DM was between three months and more than 26 years (mean, 8.50 ± 5.96 years). There were no statistical differences in the demographic variables or two biochemical measures (HbA1C and C-P levels) among the three groups, but the mean FPG in the control group was significantly lower than that in both the tai chi and qigong groups *(F*_2,84_ = 5.36, *p* = 0.027).

**Table 1:** Baseline characteristics of the study participants.

Variables	Qigong (*n* = 34)	Tai Chi (*n* = 24)	Control (*n* = 29)
Demographic			
Male sex, %	21 (62)	12 (50)	14 (48)
Age, *y*	59.71 ± 6.67	61.71 ± 6.91	58.66 ± 10.89
BMI^*a*^	25.21 ± 2.71	24.04 ± 2.98	25.69 ± 2.57
WHtR^*b*^	0.53 ± 0.05	0.52 ± 0.05	0.53 ± 0.05
Duration of T2DM, *y*	9.00 ± 6.39	9.08 ± 5.93	7.42 ± 5.49
Medications (%)			
None	8 (24)	6 (25)	13 (45)
Diaformin tablets	7 (21)	3 (13)	8 (28)
Insulin	6 (18)	5 (21)	4 (14)
Drug combination	6 (18)	6 (25)	4 (14)
Chinese medicine	2 (6)	1 (4)	0 (0)
Glimepiride tablets	3 (9)	1 (4)	0 (0)
Glipizide tablets	7 (21)	3 (13)	8 (28)
Biochemical measures			
FPG^*c*^, mmol/L	8.78 ± 3.46	8.72 ± 2.79	6.83 ± 2.80
HbA1C^*d*^, %	7.99 ± 1.66	8.20 ± 2.46	7.63 ± 1.74
C-P^*e*^, ng/mL	1.60 ± 0.81	1.37 ± 0.43	1.62 ± 1.16

Values are presented as n (%) or mean ± standard deviation.

^*a*^BMI is the weight in kilograms divided by the height squared in meters.

^*b*^WHtR is the waist circumference in centimeters divided by the height in centimeters.

^*c*^FPG is the amount of glucose present in the blood of humans and other animals before meals [29]. It is recommended in the guidelines published by the American Diabetes Association as the preferred test for diagnosing diabetes, and the lower limit for impaired fasting glucose was reduced from an FPG level of 6.1 to 5.6 mmol/L [45].

^*d*^HbA1C, which reflects the average plasma glucose concentration over 2–3 months, is weighted toward more recent levels and is useful for monitoring glycemic control in diabetic patients [46].

^*e*^C-P level is used for assessing a person’s natural insulin secretion and is used for distinguishing type 1 DM from T2DM or maturity-onset diabetes of the young [47].

BMI, body mass index; WHtR, waist-to-height ratio; T2DM, type 2 diabetes mellitus; FPG, fasting plasma glucose; HbA1C, glycated hemoglobin; C-P, C-peptide levels.

### Effects of interventions on biochemical measures

**Fig 2:**
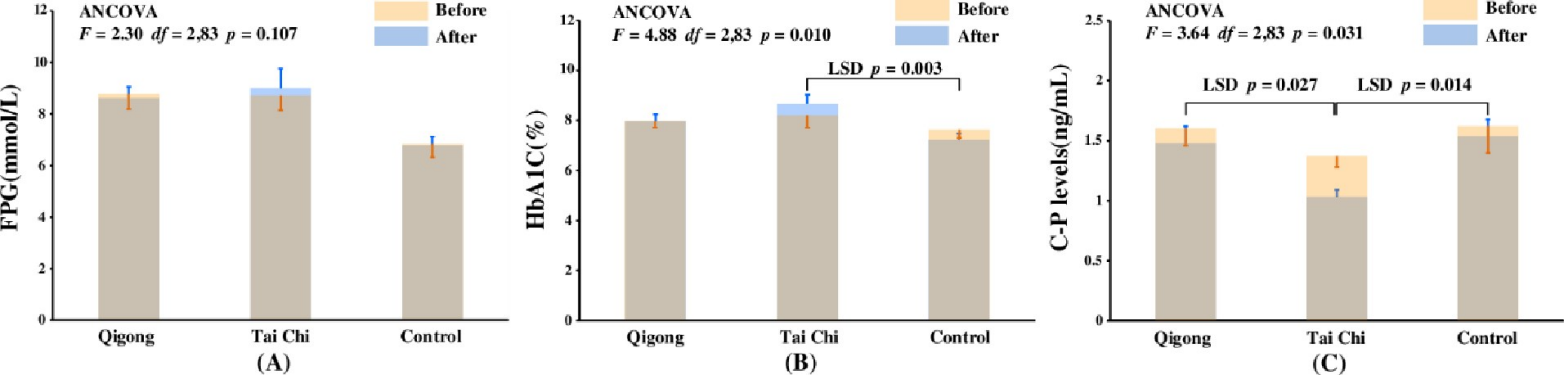
The effects of interventions on FPG (A), HbA1C (B), and C-P levels (C). FPG, fasting plasma glucose; HbA1C, glycated hemoglobin; C-P, C-peptide levels; LSD, least-significant difference; ANCOVA, analysis of covariance.

The effects of 12-week interventions on the FPG, HbA1C, and C-P levels of participants were different (Fig 2). There were no significant effects of interventions on FPG value (Fig 2A, ANCOVA, *F*_2,83_ = 2.30, *p* = 0.107). The FPG levels of participants in the control group increased, while those in the qigong group slightly decreased, and the mean FPG value of the tai chi group remain unchanged.

The interventions had significant effects on the HbA1C levels (Fig 2B, ANCOVA, *F*_2,83_ = 4.88, *p* = 0.010). The HbA1C values in the control group decreased, while those in the qigong group remained stable. There was no significant difference between the two groups (LSD, *p* = 0.084). However, HbA1C levels of participants in the tai chi intervention showed a significant increase than those in the control group (*p* = 0.003 after LSD correction).

There were also significant influences after interventions on the C-P levels (Fig 2C, ANCOVA, *F*_2,83_ = 3.64, *p* = 0.031). The C-P values of all the three groups showed a downward trend with the tai chi group showing the greatest decline. Participants in the tai chi group showed significant reduction in the C-P levels than those in the control and qigong groups when LSD correction was used for multiple comparisons (*p* = 0.014 for control vs. tai chi group and *p* = 0.027 for qigong vs. tai chi group). There were no significant differences in C-P levels between the qigong and control groups (LSD, *p* = 0.709). It is worth noting that the C-P levels of patients significantly reduced after 12 weeks of tai chi practice (paired t-test, *p* = 0.004).

### Regression analysis of the biochemical measures in the interventions

**Fig 3:**
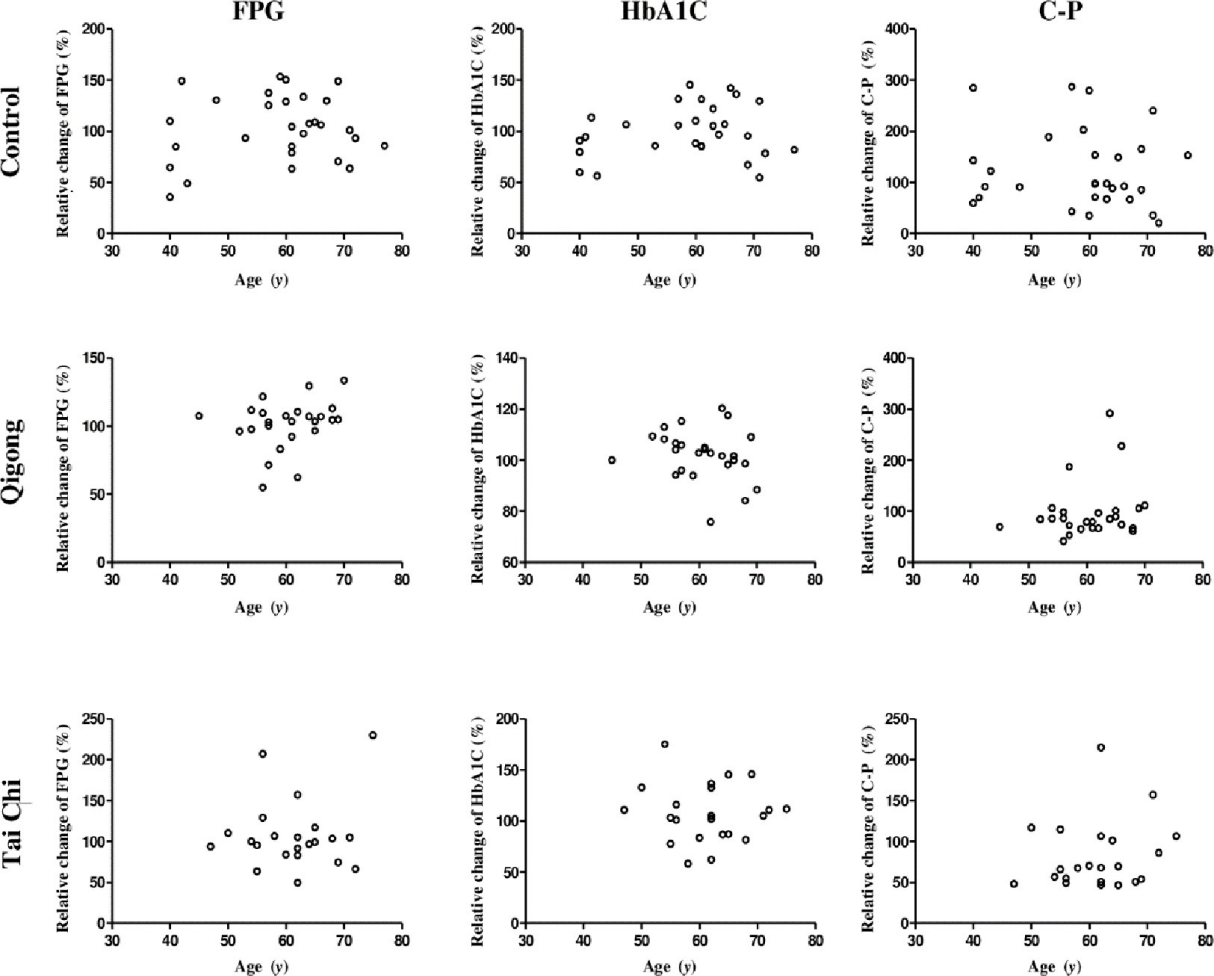
Relationship between the relative changes in the three biochemical parameters (FPG, HbA1C, and C-P level) and age (years(y)) in the control, qigong, and tai chi intervention groups for 12 weeks. FPG: fasting plasma glucose; HbA1C: glycated hemoglobin; C-P: C-peptide levels.

**Fig 4:**
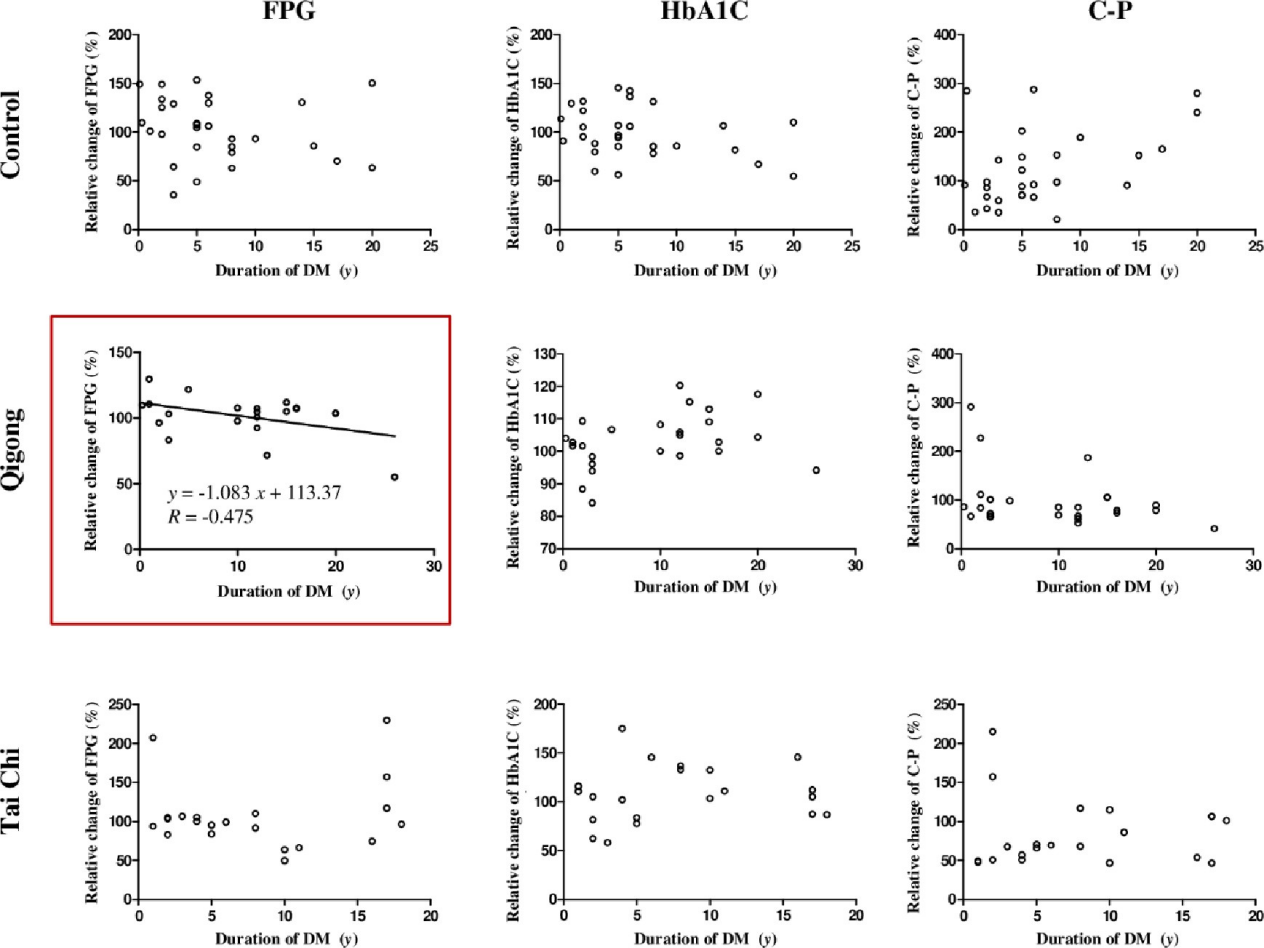
Relationship between the relative changes in the three biochemical parameters (FPG, HbA1C, and C-P level) and duration of DM (years (y)) in the control, qigong, and tai chi intervention groups for 12 weeks. FPG: fasting plasma glucose; HbA1C: glycated hemoglobin; C-P: C-peptide levels; R: Correlation coefficient; DM: diabetes mellitus.

**Fig 5:**
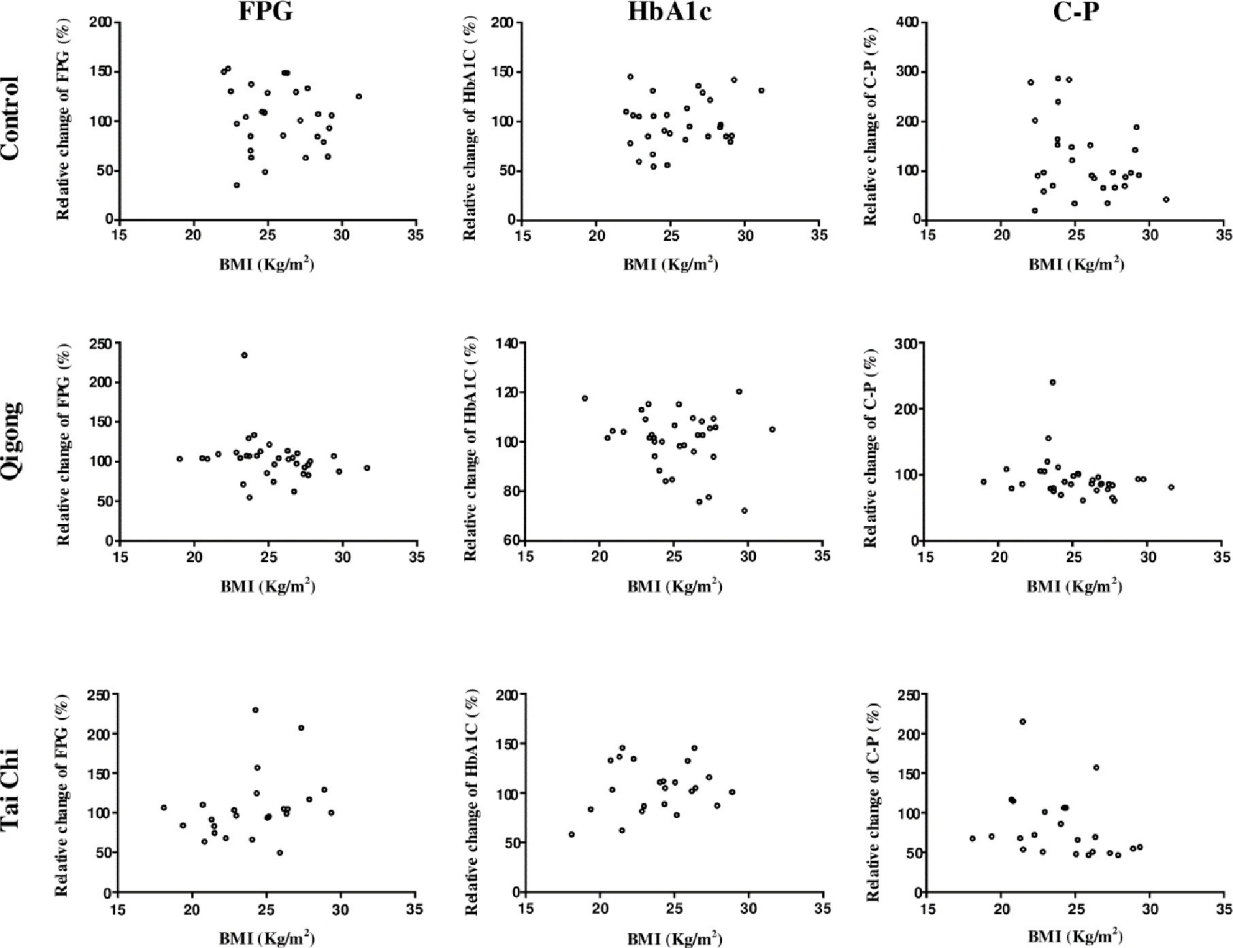
Relationship between the relative changes in three biochemical parameters (FPG, HbA1C, and C-P level) and BMI (kg/m^2^) in the control, qigong, and tai chi intervention groups for 12 weeks. FPG: fasting plasma glucose; HbA1C: glycated hemoglobin; C-P: C-peptide levels; BMI: body mass index.

**Fig 6:**
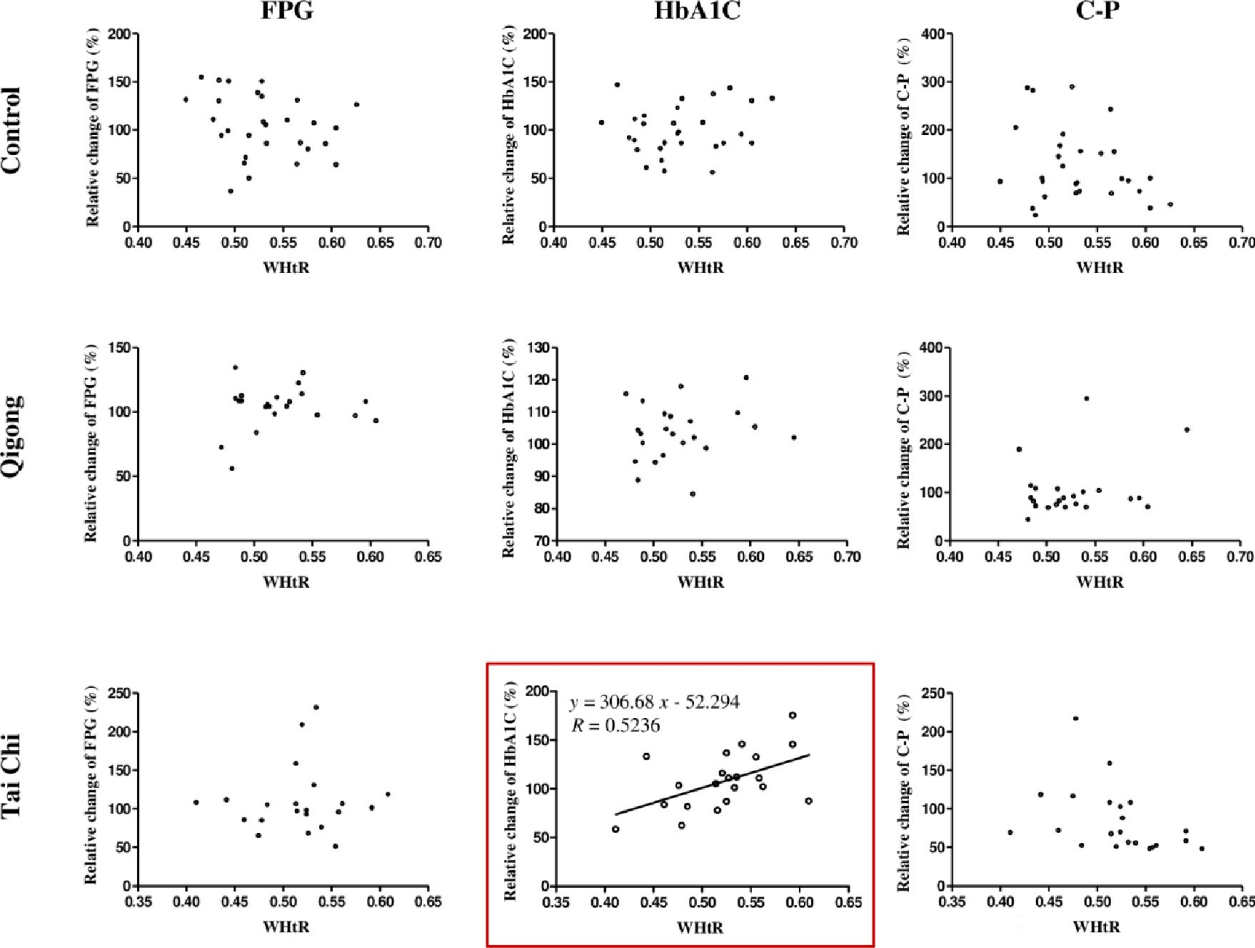
Relationship between the relative changes in the three biochemical parameters (FPG, HbA1C, and C-P level) and WHtR in the control, qigong, and tai chi intervention groups for 12 weeks. FPG: fasting plasma glucose; HbA1C: glycated hemoglobin; C-P: C-peptide levels; WHtR: waist-to-height ratio; R: correlation coefficient.

To determine the major physiological states of the participants (age, duration of DM, BMI, or WHtR) that were affected by treatment interventions, the relative changes in the three biochemical parameters from the baseline to the end of the 12-week qigong, tai chi, and control interventions were determined. The relationship between the relative changes in FPG, HbA1C, and C-P levels and age, duration of DM, BMI, and WHtR were analyzed using unary linear regression method (Figs 3–6).

In the regression analysis, the correlation coefficient R represents the degree of correlation between variable arrays x and y, and its formula is as follows:

$$R=\frac{\sum_{i=1}^{n}\left(x_{i}-\bar{x})(y_{i}-\bar{y}\right)}{\sqrt{\sum_{i=1}^{n}{\left(x_{i}-\bar{x}\right)}^{2}\sum_{i=1}^{n}{\left(y_{i}-\bar{y}\right)}^{2}}}(-1\leq r\leq 1)$$

Where, $\bar{x}=\frac{1}{n}\sum_{i=1}^{n}x_{i}$and $\bar{y}=\frac{1}{n}\sum_{i=1}^{n}y_{i}$. The closer |R| is to 1, the stronger is the linear correlation between the variable arrays *x* and *y*. *R* >0 indicates that the two variables are positively correlated, and *R* <0 indicates negative correlation.

The results showed that the duration of DM was significantly and negatively correlated with the relative changes in FPG levels in the qigong group (*R* = −0.4750, *F*_1,23_ = 6.410, *p* = 0.019, Fig 4, red square). WHtR was significantly and positively correlated with the relative changes in HbA1C levels in the tai chi intervention *(R* = 0.5236, *F*_1,21_ = 7.552, *p* = 0.012; Fig 6, red square), whereas the other physiological states of the participants were not significantly correlated with the relative changes in the biochemical parameters in the three interventions. The two standard linear regression equations were *y* = −1.083*x* + 113.37 (*y* is ΔFPG and *x* is the duration of DM) and *y* = 306.68*x* − 52.294 (*y* is ΔHbA1C and *x* is WHtR). Thus, the patients in the qigong group with shorter duration of DM reached a relatively higher mean FPG change than those in the tai chi and control group. In contrast, the patients in the tai chi group with lower mean WHtR achieved relatively higher mean HbA1C change. Age and BMI were not significantly correlated with the relative changes in the three biochemical parameters.

## Discussion

Qigong exercise had no obvious direct regulation effect on blood glucose in patients with T2DM, which may be achieved by improving the physiological functions of the internal organs for reducing blood glucose levels. In contrast, tai chi intervention had a poor effect on blood glucose regulation in patients with T2DM, which is reflected by the upward trend in HbA1C and a significant decrease in C-P levels. For patients with higher WHtR, the effect of blood glucose control was worse. Our study suggests that targeted qigong exercise may have a better interventional effect on patients with T2DM.

FPG is the most commonly used indicator of diabetes, representing the secretion of basal insulin [48]. Although FPG levels showed a downward trend after the 12-week qigong intervention, there was no significant effect on FPG levels both before and after training when compared with tai chi and the control group. In addition, HbA1C, which is the product of the binding of hemoglobin in red blood cells to blood glucose, commonly indicates glycemic control of patients during the preceding 8–12 weeks [49]. In our study, HbA1C levels were maintained stable for a long time by practicing qigong while patients were under medication, indicating that the long-term effect of blood glucose regulation was stable but did not improve significantly. C-P levels accurately reflect the secretion of endogenous insulin [50] and its determination is of great significance for the classification and diagnosis of diabetes as well as the pathogenesis of diabetes [51]. In our study, C-P levels slightly decreased in the qigong group, but there was no significant difference before and after the intervention. In addition, C-P levels can be maintained within the normal range through medication, indicating that the ability to produce insulin was not completely lost in patients, probably owing to insulin resistance [52]. Our results suggested that the qigong intervention did not significantly change endogenous insulin secretion, and its effect on blood glucose control was not significant.

One of our most salient findings was the significant negative correlation between the duration of DM and the relative changes in FPG levels at the end of the 12-week qigong intervention. In other words, a longer duration of DM was associated with more reduction in FPG levels in the qigong group. This may be because of the characteristics of qigong itself. Qigong involves a series of movements and breathing exercises selected and arranged based on the meridian theory of traditional Chinese medicine, aiming at the characteristics of diabetic patients. It is known that the longer the duration of DM, the greater the damage to the viscus [53]. The practice of qigong does not regulate blood sugar by changing insulin secretion but by training the internal organs specifically to enhance and repair the visceral functions, which improve the physiological conditions and achieve the effect of controlling blood sugar. There are also some studies on the intervention effect of Chinese fitness qigong—BaDuanJin for diabetes, which believes that each movement of qigong is targeted to regulate the related viscera [54], and autonomic viscera massage is performed through breathing exercises to improve pancreatic function [55]. These results are consistent with our findings. Furthermore, the mental exercise in qigong is used to regulate emotions and functional activities of the cerebral cortex of patients for achieving blood glucose regulation [56, 57]. In contrast, this may be owing to the irregular medication of patients with initial illness who are trying to control blood glucose with different drugs, while patients with a long history of diabetes are treated with stable drugs more regularly. Therefore, the blood glucose control effect of qigong exercise in patients with long-term disease is more reliable, which can more accurately reflect the therapeutic effect of qigong on DM. This finding on the biochemical parameters of patients with T2DM adds to the T2DM intervention research [20, 22–24, 58–60] by revealing the effectiveness of qigong for the prevention and treatment of T2DM, and more importantly, by reducing the sensitivity of insulin secretion to plasma glucose by exercise intervention [60].

Similarly, after 12 weeks of tai chi exercise intervention, FPG levels showed an upward trend, but there was no significant impact on FPG before and after training when compared with the other two groups. The tai chi group had an upward trend for HbA1C levels, while the control group showed a downward trend. There was no significant difference in HbA1C levels before and after tai chi exercise. It is worth noting that the change in HbA1C levels between the tai chi group and the control group, which was significantly different, could be by chance, implying that tai chi had no effect on HbA1C levels when compared with the control group. However, the C-P levels of patients were significantly reduced after 12-week tai chi practice, during which, the participants in the tai chi group also showed a significant reduction in the C-P levels than those in the control and qigong groups. This result suggests that the practice of tai chi may lead to long-term unstable blood sugar control, at least with the risk of deterioration [61]; this may be related to the mechanisms and characteristics of tai chi exercises. Tai chi is a systematic and complete set of martial arts with fixed sets of movements that were created for fighting on the battlefield. It is a full-body exercise that focuses more on limb movements and less on the internal viscera. In addition, it was cautiously hypothesized that tai chi exercise might lead to the destruction of islet cell storage and insulin secretion, and there was a certain risk of converting type 2 diabetes into type 1 diabetes based on the significant decrease in C-P level after tai chi intervention.

In the tai chi group of our study, the relative changes in HbA1C levels increased significantly with the increase in WHtR, presenting a positive correlation. This result indicates that centrally obese patients have higher HbA1C levels after tai chi exercise, resulting in the instability of long-term blood glucose control. Therefore, tai chi may pose a risk for people with central obesity (high WHtR), or it may not be effective for people with central obesity. The reason for this phenomenon may be different from the purpose and characteristics of tai chi and qigong exercises. Tai chi is created for fighting and winning on the battlefield and is gradually developed in the constant practice of skill and attack. The advantages of tai chi exercise, which emphasizes control over one’s displacement of body mass, postural alignment, and range of motion of the joints and muscles of the body, are more suitable for the treatment of diseases such as fibromyalgia [62] and fall injuries [33]. Some studies have also reported that tai chi can consume heat energy and promote the decomposition of sugar, which is conducive to weight loss in obese patients with T2DM and can reduce insulin resistance and increase insulin sensitivity [63]. Particularly, an investigation in Malaysia showed that long-term tai chi exercise could reduce fat accumulation, lower body fat percentage, decrease the waist-to-hip ratio, and fundamentally reduce diabetes caused by obesity [64]. However, tai chi exercise did not have a good effect on patients who failed to lose weight and lower their WHtR through tai chi practice. This may be owing to a variety of factors, such as the physical condition and dietary habits of our participants. The participants in our study were located in the Jiaozuo City of Henan Province, the north of China, and are generally stout, mainly eat wheat, and most of them suffer from diabetes caused by central obesity. The participants are less likely to slim down by practicing tai chi compared with non-obese people in the South. It was also reported that tai chi practice for diabetes mainly improved glucose and lipid metabolism of patients by consuming physical energy, reducing body weight, and promoting blood circulation [65]. Tai chi, which focuses more on physical exercise and less on the internal viscus, may not have a good effect on chronic T2DM, while the fitness qigong focuses more on the training of the internal viscus for improving the health level of patients by enhancing their own physiological functions.

Our study has some limitations. Our study was not a double-blind design, and this would have required the use of sham tai chi and qigong, for which no validated approach currently exists. Furthermore, the treatment effect that was delivered only by a single tai chi or qigong master at a single center was analyzed, which potentially limits the generalizability of our results. Finally, participants were followed up for only 12 weeks; however, there were no statistical reductions in the two biochemical measures (FPG and HbA1C); the C-P levels significantly decreased in the tai chi group than in the control and qigong groups. Hence, further prospective studies on the long-term effectiveness of qigong and tai chi in patients with T2DM needs to be conducted. Patients' dynamic measurements needs to be monitored for a long time because of the characteristics of chronic and metabolic syndrome of T2DM for evaluating yearly variations of organ damage markers in different interventions and for analyzing the associated risk factors. Furthermore, the difference in the interventional efficacy can be compared among different exercises with 6–12 months of follow-up in the future. Since qigong and tai chi are traditional Chinese martial arts, which have complex mind-body integration with a variety of active elements, such as social support, relaxation, and cognitive behavioral aspects, further studies on the improvement of qigong and tai chi for formulating an effective exercise prescription for T2DM treatment needs to be performed.

In conclusion, this single-blinded, randomized controlled trial found that there was no significant difference among the three different types of interventions for their effects on controlling diabetes. This may be due to a number of factors and limitations leading to the possible underpowered study, such as small sample size, short duration, non- guided home exercise, intensity of practice or follow-up. The difference analysis of treatment effects on patients with T2DM between qigong and tai chi may require long-term follow-ups and investigation from several aspects, which is also the focus of our research in the future.

## Supporting information

S1 Raw data (RAR)

S1 Checklist CONSORT 2010 checklist of information to include when reporting a randomised trial*. (DOC)

S1 File (DOCX)

S2 File (DOCX)

## References

[1] Mukhtar Y, Galalain A, Yunusa UJEJoB. A modern overvİew on dİabetes mellİtus: a chronic endocrine disorder. Eur J Biol. 2019;4(1): 1–14.

[2] Kitabchi AE, Umpierrez GE, Miles JM, Fisher JN. Hyperglycemic crises in adult patients with diabetes. Diabetes Care. 2009;32: 1335–1343. 10.2337/dc09-9032 19564476 PMC2699725

[3] Johnson RJ, Nakagawa T, Sanchez-Lozada LG, Shafiu M, Sundaram S, Le M, et al Sugar, uric acid, and the etiology of diabetes and obesity. Diabetes. 2013;62: 3307–3315. 10.2337/db12-1814 24065788 PMC3781481

[4] Abajobir AA, Abate KH, Abbafati C, Abbas KM, Abd-Allah F, Abdulkader RS, et al Global, regional, and national incidence, prevalence, and years lived with disability for 328 diseases and injuries for 195 countries, 1990–2016: a systematic analysis for the Global Burden of Disease Study 2016. Lancet. 2017;390: 1211–1259. 10.1016/S0140-6736(17)32154-2 28919117 PMC5605509

[5] Gardner DG, Shoback D. Greenspan`s Basic & Clinical Endocrinology. 9th ed China: McGraw-Hill Medical; 2017.

[6] Williamson RT. Causes of diabetes. Practitioner. 2009; 253: 37 19517685

[7] Tripathy B, Chandalia HB. RSSDI textbook of diabetes mellitus. 2nd ed India: JP Medical Ltd; 2012.

[8] Tfayli H, Arslanian S. Pathophysiology of type 2 diabetes mellitus in youth: the evolving chameleon. Arq Bras Endocrinol Metabol. 2009;53: 165–174. 10.1590/s0004-27302009000200008 19466209 PMC2846552

[9] Imperatore G, Boyle JP, Thompson TJ, Case D, Dabelea D, Hamman RF, et al Projections of type 1 and type 2 diabetes burden in the U.S. population aged <20 years through 2050: dynamic modeling of incidence, mortality, and population growth. Diabetes Care. 2012;35: 2515–2520. 10.2337/dc12-0669 23173134 PMC3507562

[10] Bhardwaj S, Misra A, Khurana L, Gulati S, Shah P, Vikram NK. Childhood obesity in Asian Indians: a burgeoning cause of insulin resistance, diabetes and sub-clinical inflammation. Asia Pac J Clin Nutr. 2008;17: 172–175. 18296330

[11] Misra A, Chowbey P, Makkar BM, Vikram NK, Wasir JS, Chadha D, et al Consensus statement for diagnosis of obesity, abdominal obesity and the metabolic syndrome for Asian Indians and recommendations for physical activity, medical and surgical management. J Assoc Physicians India. 2009;57: 163–170. 19582986

[12] Leutholtz BC, Ripoll I. Exercise and Disease Management. 2nd ed Boca Raton: CRC Press; 2011.

[13] Zaccardi F, Webb DR, Yates T, Davies MJ. Pathophysiology of type 1 and type 2 diabetes mellitus: a 90-year perspective. Postgrad Med J. 2016;92: 63–69. 10.1136/postgradmedj-2015-133281 26621825

[14] Cash JC, Glass CA. Family practice guidelines. 4th ed New York: Springer Publishing Company; 2017.

[15] Maruthur NM, Tseng E, Hutfless S, Wilson LM, Suarez-Cuervo C, Berger Z, et al Diabetes medications as monotherapy or metformin-based combination therapy for type 2 diabetes: a systematic review and meta-analysis. Ann Intern Med. 2016; 164(11): 740–751. 10.7326/M15-2650 27088241

[16] Saenz A, Fernandez-Esteban I, Mataix A, Ausejo M, Roque M, Moher D. Metformin monotherapy for type 2 diabetes mellitus. Cochrane Database Syst Rev. 2005;20: CD002966 10.1002/14651858.CD002966.pub3 16034881

[17] Meng D, Wei Z, Kejian L, Xianhai C. Effectiveness of t'ai chi and qigong on chronic obstructive pulmonary disease: a systematic review and meta-analysis. J Altern Complement Med. 2014;20: 79–86. 10.1089/acm.2013.0087 23961940 PMC3924809

[18] AJ K, CJ B. Oral antidiabetic agents: current role in type 2 diabetes mellitus. Drugs. 2005;65: 385–411. 10.2165/00003495-200565030-00005 15669880

[19] Perera DP, Silva REE, De, Perera WLSP. Knowledge of diabetes among type 2 diabetes patients attending a primary health care clinic in Sri Lanka. East Mediterr Health J. 2013;19: 644–648. 24975310

[20] Zinman B, Ruderman N, Campaigne BN, Devlin JT, Schneider SH; American Diabetes Association. Physical activity/exercise and diabetes mellitus. Diabetes Care. 2003;26: S73 10.2337/diacare.26.2007.s73 12502622

[21] Wayne PM, Kaptchuk TJ. Challenges inherent to t'ai chi research: part II-defining the intervention and optimal study design. J Altern Complement Med. 2008;14: 191–197. 10.1089/acm.2007.7170b 18446928

[22] Lee MS, Jun JH, Lim HJ, Lim HS. A systematic review and meta-analysis of tai chi for treating type 2 diabetes. Maturitas. 2015;80: 14–23. 10.1016/j.maturitas.2014.09.008 25449822

[23] Youngwanichsetha S, Phumdoung S, Ingkathawornwong T. The effects of tai chi qigong exercise on plasma glucose levels and health status of postpartum Thai women with type 2 diabetes. Focus Altern Complement Ther. 2013;18: 182–187.

[24] Yeh SH, Chuang H, Lin LW, Hsiao CY, Wang PW, Yang KD. Tai chi chuan exercise decreases A1C levels along with increase of regulatory T-cells and decrease of cytotoxic T-cell population in type 2 diabetic patients. Diabetes Care. 2007;30: 716–718. 10.2337/dc06-1507 17327347

[25] Yeh SH, Chuang H, Lin LW, Hsiao CY, Wang PW, et al Regular Tai Chi Chuan exercise improves T cell helper function of patients with type 2 diabetes mellitus with an increase in T-bet transcription factor and IL-12 production. Br J Sports Med. 2009;43: 845–850. 10.1136/bjsm.2007.043562 18385192

[26] Yang K, Chang W, Chuang H, Yeh S. Effects of tai chi chuan on immune and inflammatory makers of elders with and without diabetes. Innov Aging. 2017;1: 256–257.

[27] Liu T. Chinese medical qigong. 3rd ed China: Singing Dragon; 2010.

[28] Lee MS, Chen KW, Choi TY, Ernst E. Qigong for type 2 diabetes care: a systematic review. Complement Ther Med. 2009;17: 236–242. 10.1016/j.ctim.2009.05.001 19632552

[29] Wasserman DH. Four grams of glucose. Am J Physiol Endocrinol Metab. 2009;296: E11–E21. 10.1152/ajpendo.90563.2008 18840763 PMC2636990

[30] Charles MA, Balkau B, Vauzelle-Kervröedan F, Thibult N, Eschwege E. Revision of diagnostic criteria for diabetes. Lancet. 1996;348: 1657–1658. 10.1016/S0140-6736(05)65719-4 8962002

[31] Expert Committee on the Diagnosis and Classification of Diabetes Mellitus. Report of the Expert Committee on the Diagnosis and Classification of Diabetes Mellitus. Diabetes Care. 1997;20: 1183–1197. 10.2337/diacare.20.7.1183 9203460

[32] Resnick Helaine E. New diagnostic criteria for diabetes mellitus. Bmj Clinical Research. 1999;318: 531–531. 10024269 PMC1114974

[33] Li F, Harmer P, Fisher KJ, Mcauley E, Chaumeton N, Eckstrom E, et al Tai Chi and fall reductions in older adults: a randomized controlled trial. J Gerontol A Biol Sci Med Sci. 2005;60: 187–194. 10.1093/gerona/60.2.187 15814861

[34] Pfeiffer E. A short portable mental status questionnaire for the assessment of organic brain deficit in elderly patients. J Am Geriatr Soc. 1975;23: 433–441. 10.1111/j.1532-5415.1975.tb00927.x 1159263

[35] Guide map. Available online: https://baike.baidu.com/item/导引图/532283.

[36] Xun X, Ling Jian Zi. Available online: https://baike.baidu.com/item/灵剑子/6103356.

[37] Huashan Rock Art. Available online: https://baike.baidu.com/item/花山岩画.

[38] Chen Z. Chen style Tai Chi Quan book. Beijing: Peoples Sports Press; 2009.

[39] Kramer MK, Kriska AM, Venditti EM, Miller RG, Brooks MM, Burke LE, et al Translating the diabetes prevention program: a comprehensive model for prevention training and program delivery. Am J Prev Med. 2009;37: 505–511. 10.1016/j.amepre.2009.07.020 19944916

[40] Williams LB, Sattin RW, Dias J, Garvin JT, Marion L, Joshua T, et al Design of a cluster-randomized controlled trial of a diabetes prevention program within African-American churches: The fit body and soul study. Contemp Clin Trials. 2013;34: 336–347. 10.1016/j.cct.2013.01.002 23354313 PMC3594654

[41] Lindström J, Louheranta A, Mannelin M, Rastas M, Salminen V, Eriksson J, et al The Finnish Diabetes Prevention Study (DPS): Lifestyle intervention and 3-year results on diet and physical activity. Diabetes Care. 2003;26: 3230–3236. 10.2337/diacare.26.12.3230 14633807

[42] Ji EL, Ji WL, Fujii T, Fujii N, Choi JW. The ratio of estimated average glucose to fasting plasma glucose level is superior to glycated albumin, hemoglobin A1c, fructosamine, and GA/A1c ratio for assessing β-cell function in childhood diabetes. Biomed Res Int. 2014;2014: 370790 10.1155/2014/370790 25013775 PMC4071783

[43] Labocha MK, Heidi S, Hayes JP. Which body condition index is best? Oikos. 2014;123: 111–119.

[44] Stefan N, Kantartzis K, Machann J, Schick F, Häring HU. Global trends in body-mass index–Authors' reply. Lancet. 2011;377: 1917–1918. 10.1016/S0140-6736(11)60805-2 21641474

[45] American Diabetes Association. Diagnosis and classification of diabetes mellitus. 2014;37(Supplement 1): S81–S90. 10.2337/dc14-S081 24357215

[46] Tahara Y, Shima K. Kinetics of HbA1c, glycated albumin, and fructosamine and analysis of their weight functions against preceding plasma glucose level. Diabetes Care. 1995;18: 440–447. 10.2337/diacare.18.4.440 7497851

[47] Jorge AJ, Freire MD, Ribeiro ML, Fernandes LC, Lanzieri PG, Jorge BA, et al Utility of B-type natriuretic peptide measurement in outpatients with heart failure with preserved ejection fraction. Rev Port Cardiol. 2013;32: 647–652. 10.1016/j.repc.2012.10.019 23910641

[48] Moon JS, Ha KS, Yoon JS, Lee HW, Lee HC, Won KCJAD. The effect of glargine versus glimepiride on pancreatic β-cell function in patients with type 2 diabetes uncontrolled on metformin monotherapy: open-label, randomized, controlled study. Acta Diabetol. 2014;51: 277–285. 10.1007/s00592-013-0553-z 24445656

[49] Song MS, Song KH, Ko SH, Ahn YB, Kim JS, Shin JH, et al The long-term effect of a structured diabetes education program for uncontrolled type 2 diabetes mellitus patients-a 4-year follow-up. J Korean Diabetes Assoc. 2005;29: 140–150.

[50] Rendell Medicine MJAoI. Endogenous insulin secretion measured by c-peptide in maturity-onset diabetes controllable by diet alone. Arch Intern Med. 1981;141: 1617–1622. 7030247

[51] Landin-Olsson M, Nilsson KO, Lernmark A, Sundkvist GJD. Islet cell antibodies and fasting C-peptide predict insulin requirement at diagnosis of diabetes mellitus. Diabetologia. 1990;33: 561–568. 10.1007/BF00404145 2253834

[52] Ohkura T, Shiochi H, Fujioka Y, Sumi K, Yamamoto KJCD. 20/(fasting C-peptide x fasting plasma glucose) is a simple and effective index of insulin resistance in patients with type 2 diabetes mellitus: a preliminary report. Cardiovasc Diabetol. 2013;12: 21 10.1186/1475-2840-12-21 23339473 PMC3608161

[53] Gomez-Marcos MA, Recio-Rodríguez JI, Patino-Alonso MC, Cristina AC, Leticia GS, Emiliano RS, et al Yearly evolution of organ damage markers in diabetes or metabolic syndrome: data from the LOD-DIABETES study. Cardiovasc Diabetol. 2011;10: 90 10.1186/1475-2840-10-90 21999369 PMC3214163

[54] Wu YC, Wei QB. Observation of the clinical efficacy of BaDuanJin adjuvant treatment of type 2 diabetes. Chin J Gerontol. 2015; 000(018): 5218–5219 (*in Chinese*).

[55] Wu YM, Lin KL, Chen RF. Study on the effect of BaDuanJin exercise combined with health education on blood glucose intervention in 175 patients with sub-health state of diabetes. Chin Prim Health Care. 2008;02: 80–82 (*in Chinese*).

[56] Geng T. Recent research on fitness qigong BaDuanJin. GANSU Chin Med. 2008; (01): 10–12 (*in Chinese*).

[57] Fitness Qigong Management Center of the State Sports General Administration. fitness qigong. BaDuanJin. 1st ed China: Foreign Languages Press; 2003 (*in Chinese*).

[58] Yeh S, Chuang H, Lin L, Hsiao C, PW W, RT L. Regular Tai Chi Chuan exercise improves T cell helper function of patients with type 2 diabetes mellitus with an increase in T-bet transcription factor and IL-12 production. Br J Sports Med. 2009; 43: 845–850. 10.1136/bjsm.2007.043562 18385192

[59] Ruchat SM, Mottola MF. The important role of physical activity in the prevention and management of gestational diabetes mellitus. Diabetes Metab Res Rev. 2013; 29: 334–346. 10.1002/dmrr.2402 23436340

[60] Huffman FG, Vaccaro JA. Physical activity, type 2 diabetes, and ethnicity: recent findings and implications. Am J Lifestyle Med. 2013;7: 104–114.

[61] Lin CL, Lin CP, Lien SYAJHLZZtJoN. The effect of tai chi for blood pressure, blood sugar, blood lipid control for patients with chronic diseases: a systematic review. 2013; 60: 69–77. 10.6224/JN.60.1.69 23386527

[62] Wang C, Schmid CH, Rones R, Kalish R, Yinh J, Goldenberg DL, et al A randomized trial of tai chi for fibromyalgia. N Engl J Med. 2010; 363: 743–754. 10.1056/NEJMoa0912611 20818876 PMC3023168

[63] Kan Y, Zhao Y, Shao H. Effect of tai chi exercise on insulin sensitivity in obese patients with type 2 diabetes mellitus. JILIN Chin Med. 2004; 024: 11–11 (*in Chinese*).

[64] Xue GY, Chen QY, Li X, Chen Y. Formulation and implementation of quantitative exercise prescription for diabetes mellitus. Sports Sci Technol. 2009;30: 45–49 (*in Chinese*).

[65] Li HC, Qiu Y, Tie Y. Effect of Chen-style tai chi on blood biochemical indexes and cardiopulmonary function on elderly patients with Type 2 Diabetes Mellitus. Chin J Gerontol. 2015;35: 1293–1294 (*in Chinese*).

