# Supplementary material for: Effects of fitness qigong and tai chi on middle-aged and elderly patients with type 2 diabetes mellitus
Source: PLoS One. 2020 Dec 17;15(12):e0243989. doi: 10.1371/journal.pone.0243989 (PMC7746158; doi:10.1371/journal.pone.0243989)
Supplement: S1 File — (DOCX) [file pone.0243989.s003.docx]

**研究计划书**

**Research Protocol**

**（版本号：201704001）**

**项目名称： 健身气功·慢性糖尿病干预处方的研究**

**负责人： 司红玉、王振龙**

**承担单位： 郑州大学**

**电子邮箱：**  [2082370618@qq.com](mailto:2082370618@qq.com)

目录

[一、诚信声明 2](#_Toc531365617)

[二、研究题目 2](#_Toc531365618)

[三、研究计划书版本号 2](#_Toc531365619)

[四、经费来源 2](#_Toc531365620)

[五、研究事项流程图 2](#_Toc531365621)

[六、研究背景 3](#_Toc531365622)

[七、研究目的 3](#_Toc531365623)

[八、纳入和排除标准 4](#_Toc531365624)

[九、设计方案 4](#_Toc531365625)

[十、样本量估算 5](#_Toc531365626)

[十一、随机和隐蔽分组的方法 5](#_Toc531365627)

[十二、盲法 5](#_Toc531365628)

[十三、测量指标 5](#_Toc531365629)

[十四、对于参试者有效性的定义 6](#_Toc531365630)

[十五、不良事件和不良反应的定义、鉴别和管理制度 7](#_Toc531365631)

[十六、伦理考量 7](#_Toc531365632)

[十七、受试者招募 7](#_Toc531365633)

[十八、参试者一般信息的收集 8](#_Toc531365634)

[十九、基线指标和观测项目 8](#_Toc531365635)

[二十、标准操作规程 8](#_Toc531365636)

[二十一、统计分析方法 8](#_Toc531365637)

[二十二、参试者管理制度 9](#_Toc531365638)

[二十三、标本管理制度 9](#_Toc531365639)

[二十四、数据管理制度包括数据采集、源数据及其文件的管理、采集和录入（记录）人员、核对制度 9](#_Toc531365640)

[二十五、数据安全与监察委员会的组成和工作职责 10](#_Toc531365641)

[二十六、研究团队 10](#_Toc531365642)

[二十七、知识产权 11](#_Toc531365643)

[二十八、发表计划 11](#_Toc531365644)

[二十九、原始数据共享计划 11](#_Toc531365645)

[三十、试验结束后对参试者的治疗和管理 11](#_Toc531365646)

# 一、诚信声明

本课题组郑重声明，本研究操作过程均严格按照研究计划严格执行，认真诚实记录实验数据。本课题所产生的一切研究成果，包括技术标准、专利等相关知识产权归属课题组所有。课题组完全意识到本声明应承担的法律责任。

# **二、研究题目**

健身气功·慢性糖尿病干预处方的研究

# 三、研究计划书版本号

版本号：201704001

# 四、经费来源

国家体育总局健身气功管理中心（编号QG2017019）

# 五、**研究事项流程图**

| **时间安排** | **研究任务** | **主要目标** |
| --- | --- | --- |
| 起2017.04 | 召开课题启动会，协调各单位的分工合作，进行项目培训、伦理审查申请。制定合格被试标准，确定检测指标，选择心理量表，制定操作手册，进行研究人员人才培养。 | 招募被试者，进行初步的信息审查，检查检测被试的身体状况，对不符合标准者及时排出，更新被试。 |
| 止2017.06 |  |  |
| 起2017.07 | 第一次课题总结论证会，组织课题培训，进行随机单盲分组，进行基本数据的采集和记录，将患者资料录入数据库。 | 筛查和确定被试者，招募T2DM患者103名，完成所有指标测试，整理相关数据，进行初步分析与评估。 |
| 止2017.10 |  |  |
| 起2017.11 | 按照分组固定教授被试体育运动（太极、气功和拉伸运动）。进行中期会议，总结和协调实施方案中出现的问题，进行人才培养。 | 将患者随机分成3组（太极、气功、对照），进行为期12周的干预习练，每周固定时间习练5次，每次1小时。对习练过程中出现的异常情况进行及时的调整。 |
| 止2018.01 |  |  |
| 起2018.02 | 梳理研究资料，对于研究数据进行初步分析，聘请专家进行学术交流。 | 完成干预习练后1周的患者各项生化指标数据检测和调查问卷，整理数据，综合分析所有参数，找出健身气功功法处方锻炼对习练者的病程疗效、生理指标提升和心智改善的作用。 |
| 止2018.05 |  |  |
| 起2018.06 | 召开数据挖掘讨论会议，课题研究成果论证会议，集中撰写结项报告和学术论文。 | 整理检测数据资料，综合分析与评估健身气功功法处方对糖尿病患者的作用，撰写学术论文。整理和完善研究报告。 |
| 止2018.12 |  |  |

# 六、研究背景

糖尿病是一种慢性病，当胰腺产生不了足够的胰岛素或者人体无法有效地利用所产生的胰岛素时，就会出现糖尿病。据国际糖尿病联盟（The International Diabetes Federation ，IDF），2017年全球糖尿病地图（第8版）报告显示：如今，中国糖尿病患者约为1.21亿，已经成为影响人类幸福生活的主要慢性病之一。糖尿病是失明、肾衰竭、心脏病发作、中风和下肢截肢的主要病因。运动治疗是现代糖尿病管理的重要内容之一。世界卫生组织（WHO）提出：健康饮食、经常锻炼身体、保持正常体重和避免使用烟草，可预防二型糖尿病或推迟其发病。糖尿病可得到治疗，其并发症可通过膳食、身体活动、服药和定期筛查和治疗得到避免或延迟出现。因此，通过运动干预改善糖尿病患者的生存质量，已经被广泛采用。

太极拳是我国最优秀的民族传统体育项目之一。太极拳以中国传统儒、道哲学中的太极、阴阳辩证理念为核心思想，集颐养性情、强身健体、技击对抗等多种功能为一体，是一种注重呼吸和意念、柔和、缓慢、轻灵的传统拳术。世界卫生组织(WHO)已将太极拳列为心脏复健运动项目。太极拳作为运动干预手段对于慢性病有着积极的效果。

健身气功作为传统体育养生项目之一，由2001年6月成立的国家体育总局健身气功管理中心具体负责，是我国正式开展的第62个传统体育项目。16年来，学术成果得到持续发展：首先，以科研课题立项的形式，组成编创了由健身效果数据作为支撑的9套健身气功新功法、4套竞赛功法，随后，以它们为基础进行各领域的研究并具有一些特点：比如学科的交叉化、深入化，学练对象的多样化，研究人群的多元化。研究健身气功的养生机理及效果已成为体育科研的一个新热点。

# 七、研究目的

依据中国传统养生学的基本理论，也就是中医理论体系和疾病防治技术，以“调身”、“调息”和“调心”为核心技术，契合中医养生学思想，开展糖尿病的预防与辅助治疗的临床研究，形成可推广的、规范的慢性糖尿病气功预防和辅助治疗的运动处方，客观评价三种运动干预对于糖尿病患者的临床辅助治疗和身心效果。该项目的研究成果和技术为广泛推广和普及气功等传统健身养生体育锻炼提供一定的理论依据，培养国际化、多学科交叉人才，并以此推动中外文化交流互鉴。

# 八、纳入和排除标准

（一）纳入标准

1．确诊为2型糖尿病，病史三个月以上，年龄40—75岁。

2．过去三个月没有参与中度或重度的劳动。

3．没有不能参与锻炼的疾病或伤害。

4．神智清醒，具有正常的沟通能力，没有认知障碍。

（二）排除标准

1．I型糖尿病患者。

2．在过去六个月参与过气功或太极训练。

3．患有不能参与运动的疾病或有其他身体伤害的患者。

# 九、设计方案

以中老年2型糖尿病患者为研究对象，采用随机平行研究设计，与焦作第二人民医院合作，通过初步电话访问和医生咨询等方式初步筛选103例患者。根据纳入标准，将纳入病例随机分入气功组，太极组和对照组。气功组进行自编气功功法训练，太极组进行陈氏十八式太极拳训练，对照组进行健康教育和同等强度的拉伸训练，干预时间为12周。在干预训练开始前进行生理指标、糖尿病相关生化指标的测量，以及填写心理调查问卷测评表。12周干预结束后，再次进行生理指标、糖尿病相关生化指标的测量以及心理问卷测评表的填写。本研究的干预治疗属于探索性研究，主要目的是探索传统健身气功、太极拳对慢性糖尿病的预防与干预治疗效果。具体技术路线图如下：

# 十、样本量估算

根据以往的气功和太极对于慢性病的干预效果研究，每组样本量约为30例，共90例。按照20%的脱落率，总样本约为110例。

# 十一、随机和隐蔽分组的方法

将患者信息录入计算机，计算机随机，产生随机安排，随机方案放置在不透光的牛皮信封里。

# 十二、盲法

由于试验疗法中包含气功和太极训练，气功组采用气功训练，太极组采用太极拳锻炼，对照组进行健康教育与拉伸锻炼。因此，对于教练员以及患者不设盲法，对于其余研究人员设置盲法。

# 十三、测量指标

（一）主要疗效指标

1．空腹血糖（FPG）

2．糖化血红蛋白（HbA1C）

3．C肽反应（C-P）

（二）次要指标

1．幸福感指数量表

2．情感平衡量表

3．抑郁量表

4．SF-36表

（三）安全性指标

1．不良事件，包括干预习练造成的运动伤害或者疲劳不能坚持等情况。

上述主要指标和次要指标分别在分组后训练前进行一次检测，以及在完成12周的干预训练后再进行一次检测，检测地点在焦作第二人民医院。安全性指标，及不良事件的反应可以随时向教练反应，或者每周固定一次的医生随访。

# 十四、对于参试者有效性的定义

（一）受试者有权在临床试验的任何阶段随时退出试验。研究者有义务采取必要措施，包括主动做出让受试者退出临床试验的决定，以保障受试者的安全和权益。

（二）在研究过程中，出现以下问题，研究者应主动考虑让受试者退出试验：

1．受试者出现病情加重，继续参与不利于受试者治疗；

2．受试者依从性差，在研究干预等方面不能按照临床试验方案执行；

3．出现严重的并发症或者其他不能运动的疾病或伤害；

4．可能增加受试者风险或影响研究结果可靠性的其他情况。

（三）受试者主动退出临床试验

1．不应因此受到任何歧视或报复；

2．受试者退出试验，应被告知如何以及从何处获得其他可能的治疗方法；

3．研究者应尽可能了解受试者主动退出试验的原因，并将相关信息记录。

（四）研究者应将自己的联系方式主动告知被试者，并主动获取受试者的最新联系方式，以确保定期定时参与训练；

（五）受试者退出临床试验的相关信息应进行记录，并定期上交伦理委员会；

（六）受试者在退出试验并不意味受试者的试验数据退出试验。截止到退出节点，已获得的实验数据应作为数据库的一部分予以保留。

# 十五、不良事件和不良反应的定义、鉴别和管理制度

（一）研究者应主动与受试者充分交流、主动询问、详尽检查、审核数据等，充分收集受试者安全信息，及时准确地判断研究过程中的不良事件。

（二）不良事件可以是与研究实施期间中任何不利和意想不到的迹象、病症或伤害，无论是否考虑与研究干预相关。

（三）严重不良事件是指在试验干预期间出现的以下不良事件：包括：伤残，影响工作能力、危及生命等事件。

（四）确认不良事件后，应首先做出是否为严重不良事件的判断。普通不良事件根据实际情况，给予相应的临床处理，并进行记录。

# 十六、伦理考量

本研究的目的是检验运动处方对糖尿病的预防与辅疗效果及其对身体素质的提升作用，激发健身气功养生文化的生机与活力，以科学性增强其文化自觉与文化自信，为弘扬传统优秀文化，建设社会主义文化强国服务。本研究的实施及检测结果并不会对传统伦理道德产生冲击。

# 十七、受试者招募

（一）针对本研究试验方案和研究中心自身特点制定可行的招募策略，本研究主要针对中老年（40岁以上）2型糖尿病的患者，患者的招募主要是在2016年-2017年在焦作第二人民医院注册看病的患者数据库中选取，通过电话访问或者医生的咨询等方式进行初步合格患者的招募。

（二）受试者由专门的人员负责，以便于相关工作的管理，信息记录和进度的把握。

（三）无论采用何种招募方式，招募过程都是非强制性的。

（四）招募过程中涉及相关费用尽量清晰说明，避免使用笼统字眼，避免误会。

（五）特别注意避免以下情况的招募者

1．不在焦作居住，难以保证参与训练的患者；

2．因为各种原因，难以理解和依从本方案的受试者；

3．正在参与其他试验方案的受试者；

4．有导致医患纠纷倾向的受试者。

# 十八、参试者一般信息的收集

收集的参试者信息的内容包括：身高、体重、性别、年龄、教育程度、腰围、糖尿病史、用药情况和其他疾病等。

# 十九、基线指标和观测项目

基线指标：身高，体重，腰围，病史，用药史，BMI，心率，收缩压，舒张压，血糖，糖化血红蛋白，C肽反应，幸福感指数，情感平衡指数，抑郁指数，SF-36测量表。

观测项目：血糖，糖化血红蛋白，C肽反应，幸福感指数，情感平衡指数，抑郁指数，SF-36测量表。

# 二十、标准操作规程

训练十二周，每周五次，每次训练一小时，十分钟的热身活动，十分钟的放松活动，四十分钟的气功、太极和同等运动强度的拉伸训练。气功组选择由郑州大学体育学院气功小组开发创编的气功功法，共有20式，预备式至第6式为功前准备，第7式至第15式为正式功法，第16式至第19式为功后整理。整套功法练习时长8-10分钟。太极组选择由陈正雷创编的经典陈氏太极十八式，共有十八个动作，完整练习一次为5-6分钟。拉伸对照组的训练主要由专业的健身教练进行的同等强度、配合运动器械的躯体拉伸运动，每个动作根据自身的承受能力连续重复10-15次，主要针对身体的伸展和协调设计的运动。

# 二十一、统计分析方法

课题组专门设立了焦作第二人民医院化验科的专业人员，负责2型糖尿病患者的生化指标（FPG、HbA1C和C-P）的检测和采集记录。并且专门成立数据记录和采集分析小组，进行试验过程中的数据记录与分析的培训，主要负责记录干预前后三组被试者的问卷调查数据记录和分析；聘请有10年以上气功、太极拳和健身教学经验的3位教练进行专业的干预习练教学，在教学过程中有专门的人员监督和统计学员的习练情况、不良反应事件和督导学员平时自己在家的训练。所有的数据经过记录采集建立数据档案，由专门的分析人员进行数据分析。

采用双因素方差分析（ANOVA）方法，设置检验为糖尿病（+/-）×功法（+/-），分析功法的疗效；配对*t*检验分析被试者学练前后的参数差异；采用logistic回归方法综合分析年龄、性别、功法等多种因素对糖尿病的疗效、生理作用和心智改善作用。

统计分析将采用SPSS 21.0统计分析软件进行计算。所有的统计检验均采用双侧检验，*p*值小于或等于0.05将被认为所检验的差别有统计意义。

# 二十二、参试者管理制度

（一）在临床试验启动前，有必要为哭啼项目的实施设立一个核心的质量管理小组，指派专门的质量管理人员。

（二）参试者应该为课题项目的实施制定本项目内部的质量管理策略，明确质量目标。

（三）研究团队

1．研究团队应保证所有的参与人员具备执行和完成试验流程的资质；

2．根据试验方案分解任务，明确每一位研究团队成员的责任分工。

3．研究小组内部建立培训机制，确保所有参研人员充分具备完成试验所需的知识和信息。

4．尽可能保证研究团队的稳定性。

# 二十三、标本管理制度

本次血糖检测均在焦作市第二人民医院完成，所有操作规范及流程符合标本采集要求。

采集：严格执行无菌技术操作规程，必须使用一次性采血针，使用后的一次性采血针不得重复使用。采血时必须一人、一针、一片（试纸），避免交叉感染。采血部位通常采用指尖两侧的末梢毛细血管，水肿、感染或硬茧的部位不宜采血。手臂下垂秒，用复合碘医用棉签擦拭采血部位，待自然干燥后进行皮肤穿刺。皮肤穿刺后，轻轻按摩手指挤出血液，置于试纸上指定区域。用干燥棉签压制采血点。

送检：由检验科抽取，抽取后立刻送检。

保管：血糖检测所采集的血液样本采集之后立即进行血糖检测，经过血糖检测之后由医院保管，由医院送往医疗垃圾处理点集中处理。

# 二十四、数据管理制度包括数据采集、源数据及其文件的管理、采集和录入（记录）人员、核对制度

（一）数据采集

患者认真填写基本情况调查表，以及研究小组成员分发的心理问卷。血糖检测结果由焦作市第二人民医院统一给出。

（二）数据的录入

由两个数据录入员分别将数据录入计算机。

（三）数据保管

纸质数据分小组分装在专门的档案袋中，放置于郑州大学体育学院（校本部）。电子数据保存在专门的硬盘中，硬盘放置于郑州大学体育学院（校本部）。

# 二十五、数据安全与监察委员会的组成和工作职责

为了保障项目的顺利实施和确保试验研究的风险最小化，项目组专门成立了数据安全与监察委员会。组长由项目主持人司红玉教授担任，主要负责全面监督和负责试验研究的数据和安全工作；数据安全副组长由王振龙教授担任，主要负责试验的设计和数据的采集和记录流程的安全和正确；试验副组长由范海生负责，主要负责气功功法、太极拳功法和健身运动的教练教学监督和动作技巧的规范性，保证干预手段的规范性、合理性和有效性。其余成员包括：专门的数据采集和分析人员、招募和随访人员、教学监督人员、医护人员、专业的化验人员从各个方面严把质量关，保证在整个试验过程中数据的安全准确、规范有效。

# 二十六、研究团队

|  | **姓 名** | **分工** |
| --- | --- | --- |
| 负责人 | 司红玉 | 组织管理 |
|  | 王振龙 | 实验设计 |
|  | 范海生 | 提供功法 |
| 主要参加人员 | 李晓媛 | 数据分析 |
|  | 陈亚民 | 数据分析 |
|  | 张义锋 | 实验测试 |
|  | 田欣茂 | 数据采集 |
|  | 李怀亮 | 教学监控 |
|  | 韩向阳 | 教学监控 |
|  | 尹宁宁 | 教学监控 |
|  | 杨天雨 | 教学监控 |
|  | 张麒 | 招募人员 |
|  | 夏寒 | 招募人员 |

# 二十七、知识产权

本项目所取得的研究成果归属研究团队共同拥有，产出的相关研究论文、专利等知识产权归属郑州大学研究团队。本着公平、公正的原则，产出的研究成果根据工作贡献的大小，作者的署名顺序由合作人协商完成，大家没有相关的利益冲突。如果协商有异议，由项目主持人公正处理决定。

# 二十八、发表计划

本项目到项目完成验收时完成研究报告一份，预计发表论文1-3篇。

# 二十九、原始数据共享计划

不公开

# 三十、试验结束后对参试者的治疗和管理

本研究只检验运动处方对糖尿病的预防与辅疗效果及其对身体素质的提升作用，比较气功和太极对于糖尿病患者的身心的干预效果，并不是对参试者进行治疗。
