# Supplementary material for: Effects of fitness qigong and tai chi on middle-aged and elderly patients with type 2 diabetes mellitus
Source: PLoS One. 2020 Dec 17;15(12):e0243989. doi: 10.1371/journal.pone.0243989 (PMC7746158; doi:10.1371/journal.pone.0243989)
Supplement: S2 File — (DOCX) [file pone.0243989.s004.docx]

**Research Protocol**

**（Version Number：201704001）**

**Project name： Study on Intervention Prescription of**

**Chronic Type 2 Diabetes using Health Qigong**

**Project leader： Si Hongyu**

**Responsible institutions： Zhengzhou University**

**Email：**  [**2082370618@qq.com**](mailto:2082370618@qq.com)

Contents

[1、Statement of Integrity 1](#_Toc35458186)

[2、Research topics 1](#_Toc35458187)

[3、Research proposal version number 1](#_Toc35458188)

[4、Source of Funding 1](#_Toc35458189)

[5、Research Matter Flowchart 1](#_Toc35458190)

[6、Research Background 3](#_Toc35458191)

[7、Research purposes 4](#_Toc35458192)

[8、Inclusion and exclusion criteria 4](#_Toc35458193)

[9、Design scheme 5](#_Toc35458194)

[10、Sample size estimation 6](#_Toc35458195)

[11、Random and covert grouping method 6](#_Toc35458196)

[12、Blind method 6](#_Toc35458197)

[13. Measurement index 7](#_Toc35458198)

[14. Definition of participant effectiveness 7](#_Toc35458199)

[15. Definition, identification and management system of adverse events and adverse reactions 8](#_Toc35458200)

[16. Ethical considerations 9](#_Toc35458201)

[17. Subject recruitment 9](#_Toc35458202)

[18. General information collection of participants 10](#_Toc35458203)

[19. Baseline indicators and observation items 10](#_Toc35458204)

[20. Standard operating procedures 10](#_Toc35458205)

[21. Statistical analysis method 11](#_Toc35458206)

[22. Participant management system 11](#_Toc35458207)

[23. Specimen management system 12](#_Toc35458208)

[24. Data management system includes data collection, management of source data and its documents, collection and entry (record) personnel and check system 13](#_Toc35458209)

[25. Composition and responsibilities of data security and supervision committee 13](#_Toc35458210)

[26. Research team 14](#_Toc35458211)

[27. Intellectual property 15](#_Toc35458212)

[28. Publication plan 15](#_Toc35458213)

[29. Original data sharing plan 16](#_Toc35458214)

[30. Treatment and management of participants after the trial 16](#_Toc35458215)

# 1、Statement of integrity

The research team solemnly declares that the research operation process is strictly implemented in strict accordance with the research plan, and the experimental data is carefully and honestly recorded. All research results produced by this topic, including technical standards, patents and other related intellectual property rights, belong to the research group. The research team is fully aware of the legal responsibilities that this statement should bear.

# 2、Research topics

Study on Intervention Prescription of Chronic Type 2 Diabetes using Health Qigong

# 3、Version number of research proposal

Version number: 201704001

# 4、Source of funding

Funded by the Health Qigong Management Center of Sports General Administration of China.(No. QG2017019)

# 5、Flowchart of study items

| **Schedule** | **Research task** | **Main target** |
| --- | --- | --- |
| Start 2017.06 | Held a project initiation meeting to coordinate the division of labor and cooperation among various units to conduct project training and ethical review applications. Formulate the standards for qualified subjects, determine the detection indicators, select psychological scales, develop operating manuals, and conduct research personnel training. | Recruit subjects, conduct a preliminary information review, check and detect the physical condition of the subjects, and discharge those who do not meet the standards in a timely manner to update the subjects. |
| Ends 2017.06 |  |  |
| Start 2017.07 | The first general meeting of subject conclusions, organized subject training, randomized single-blind grouping, collected and recorded basic data, and entered patient data into the database. | Screened and identified participants, recruited T2DM patients, completed all index tests, collated relevant data, and conducted preliminary analysis and evaluation. |
| Ends 2017.10 |  |  |
| Start 2017.11 | Participate in regular teaching of physical exercise (tai chi, qigong, and stretching) in groups. Conduct mid-term meetings, summarize and coordinate the problems in the implementation plan, and train talents. | Patients were randomly divided into 3 groups (tai chi, qigong, and control) for intervention training for 12 weeks, 5 times a week at a fixed time, 1 hour each time. Make timely adjustments to abnormal situations that occur during the practice. |
| Ends 2018.01 |  |  |
| Start 2018.02 | Comb the research data, conduct a preliminary analysis of the research data, and hire experts for academic exchanges. | One week after the completion of the intervention exercise, the patient's biochemical index data testing and questionnaires were collected and the data were collated. Comprehensive analysis of all parameters to find out the effects of Health Qigong Prescription Exercise on the course effect, physiolog-  ical index improvement and mental improvement of the trainees. |
| Ends 2018.05 |  |  |
| Start 2018.06 | Held a data mining discussion meeting, a topic research results demonstration meeting, and focused on writing a final report and academic paper. | Sort out test data, comprehensively analyze and evaluate the effect of health qigong prescription on diabetic patients, and write academic papers. Organize and improve the research report. |
| Ends 2018.12 |  |  |

# 6、Research bckground

Diabetes is a chronic disease that occurs when the pancreas does not produce enough insulin or the body cannot effectively use the insulin produced. According to the International Diabetes Federation (IDF), the 2017 Global Diabetes Map (8th Edition) report shows that now there are approximately 121 million people with diabetes in China, which has become one of the major chronic diseases that affect human happiness. Diabetes is a major cause of blindness, kidney failure, heart attack, stroke, and lower limb amputations. Exercise therapy is one of the important contents of modern diabetes management. The World Health Organization (WHO) has proposed that a healthy diet, regular exercise, maintaining a normal weight and avoiding tobacco use can prevent or delay the onset of type 2 diabetes. Diabetes can be treated, and its complications can be avoided or delayed through diet, physical activity, medication, and regular screening and treatment. Therefore, exercise intervention to improve the quality of life of patients with diabetes has been widely adopted.

Tai chi is one of the finest national traditional sports in China. Tai chi takes the tai chi and Yinyang dialectical concepts of traditional Chinese Confucianism and Taoism as its core ideas, and integrates multiple functions such as tempering, strengthening, and fighting. It is a kind of emphasis on breathing and ideas, gentle, slow, light Spiritual traditional boxing. The World Health Organization (WHO) has listed tai chi as a cardio rehabilitation program. Tai chi as a sports intervention has a positive effect on chronic diseases.

Health qigong, as one of the traditional sports health programs, is specifically responsible for the health qigong Management Center of the State General Administration of Sport established in June 2001. It is the 62nd traditional sports program officially launched in China. In the past 16 years, the academic achievements have continued to develop: First, in the form of scientific research project establishment, we have created 9 sets of fitness qigong new exercises and 4 sets of competition exercises supported by fitness effect data. Then, based on them, research in various fields has some characteristics: for example, the interdisciplinary and deepening of disciplines, the diversity of learning and training objects, and the diversity of research populations. Research on the health mechanism and effects of health qigong has become a new hotspot in sports research.

# 7、Research purposes

According to the basic theories of traditional Chinese health care, that is, the theoretical system of Chinese medicine and disease prevention and treatment technology, and take "adjusting body", "adjusting interest rate" and "adjusting heart" as the core technology, in line with the thought of traditional Chinese medicine health care, carry out clinical research on the prevention and adjuvant treatment of diabetes, form a generalizable and standardized exercise prescription for the prevention and adjuvant treatment of chronic diabetes qigong, and objectively evaluate the clinical adjuvant treatment and physical and mental effects of three kinds of exercise interventions on patients with diabetes. The research results and technology of the project provide a certain theoretical basis for the widespread promotion and popularization of traditional fitness and health exercises such as qigong, training internationalized, multidisciplinary talents, and promoting cultural exchanges between China and foreign countries.

# 8、Inclusion and exclusion criteria

（1）Inclusion criteria

1) Diagnosed as type 2 diabetes, with a history of more than three months, aged 40-75.

2) Did not participate in moderate or severe labor in the past three months.

3) There are no illnesses or injuries that cannot participate in exercise.

4) Sane, with normal communication ability, without cognitive impairment.

（2）Exclusion criteria

1) Patients with type I diabetes.

2) Participated in qigong or tai chi training in the past six months.

3) Patients with diseases that cannot participate in sports or other physical injuries.

# 9、Design scheme

Taking the middle-aged and elderly patients with type 2 diabetes as research objects, using a randomized parallel study design, cooperating with Jiaozuo Second People's Hospital, patients were selected preliminarily through telephone interview and doctor consultation. According to the inclusion criteria, the patients will be randomly divided into qigong group, tai chi group and control group. Qigong group will be trained with self-made qigong, tai chi group will be trained with Chen's 18 Style tai chi, and the control group will be trained with health education and stretching of the same intensity, the intervention time is 12 weeks. Before the intervention training, the physiological indexes and diabetes related biochemical indexes are measured, and the psychological questionnaire is filled in. After 12 weeks of intervention, physiological indexes, diabetes related biochemical indexes and psychological questionnaire will be filled in again. The intervention treatment of this study is an exploratory study, and the main purpose is to explore the effects of traditional health qigong and tai chi on the prevention and intervention of chronic diabetes. The specific technology roadmap is as follows:

# 10、Sample size estimation

According to the previous study on the intervention effect of qigong and tai chi on chronic diseases, the sample size of each group is about 30 cases, a total of 90 cases. According to the 20% shedding rate, the total sample is about 110 cases.

# 11、Random and covert grouping method

The patient information is input into the computer, and the computer randomly generates a random arrangement, the random scheme is placed in a Kraft envelope.

# 12、Blind method

As the experimental therapy includes qigong and tai chi training, qigong group adopts qigong training, tai chi Group adopts tai chi training, and the control group carries out health education and stretching exercise. Therefore, there is no blind method for coaches and patients, and blind methods for other researchers.

# 13. Measurement index

(1) Main efficacy indicators

1) Fasting blood glucose (FPG)

2) Glycated hemoglobin (HbA1c)

3) C-peptide reaction (C-P)

(2) Secondary indicators

1) Happiness index scale

2) Emotional balance scale

3) Depression scale

4) SF-36 table

(3) Safety index

1) Adverse events, including sports injury or fatigue caused by dry rehearsal.

The above main and secondary indexes were tested before the training after grouping and after 12 weeks of intervention training. The testing unit was Jiaozuo Second People's hospital. Safety indicators and adverse events can be reported to the coach at any time or followed up by the doctor once a week.

# 14. Definition of participant effectiveness

(1) Subjects have the right to withdraw from the trial at any stage of the clinical trial. Researchers are obliged to take necessary measures, including taking the initiative to withdraw the subjects from the clinical trial, in order to protect the safety and interests of the subjects.

(2) In the course of the study, the researcher should take the initiative to consider the withdrawal of the subjects in case of the following problems:

1. If the patient's condition worsens, continuing to participate in the treatment is not conducive to the patient;

2. The subjects had poor compliance and could not follow the clinical trial plan in terms of research intervention;

3. Serious complications or other diseases or injuries that can't move;

4. Other circumstances that may increase the risk of subjects or affect the reliability of research results.

(3) Subjects voluntarily withdraw from the clinical trial

1. Shall not suffer any discrimination or retaliation as a result;

2. When subjects withdraw from the trial, they should be informed how and where to obtain other possible treatment methods;

3. The researcher should try to understand the reasons for the subjects to withdraw from the trial and record the relevant information.

(4) Researchers should actively inform the subjects of their contact information, and actively obtain the latest contact information of the subjects to ensure regular and regular participation in training;

(5) The information related to the withdrawal of subjects from the clinical trial shall be recorded and submitted to the Ethics Committee regularly;

(6) The withdrawal of subjects from the trial does not mean that the trial data of subjects are withdrawn from the trial. As of the exit node, the acquired experimental data should be kept as part of the database.

# 15. Definition, identification and management system of adverse events and adverse reactions

(1) Researchers should take the initiative to fully communicate with the subjects, actively inquire, conduct detailed inspection, audit data, etc., fully collect the safety information of the subjects, and timely and accurately judge the adverse events in the research process.

(2) Adverse events can be any adverse and unexpected signs, symptoms or injuries during study implementation, whether or not considered related to study interventions.

(3) Serious adverse events refer to the following adverse events occurred during the experimental intervention: Including: disability, affecting work ability, life-threatening and other events.

(4) After confirming the adverse event, the judgment of whether it is a serious adverse event shall be made first. According to the actual situation, common adverse events were given corresponding clinical treatment and recorded.

# 16. Ethical considerations

The purpose of this study is to test the effect of exercise prescription on prevention and treatment of diabetes mellitus and its promotion on physical quality, stimulate the vitality and vitality of health culture of fitness qigong, scientifically enhance its cultural consciousness and cultural self-confidence, and serve for promoting traditional excellent culture and building a socialist cultural power. The implementation and test results of this study will not have an impact on traditional ethics.

# 17. Subject recruitment

(1) According to the experimental scheme and the characteristics of the research center, feasible recruitment strategies are developed. This study is mainly aimed at the elderly (over 40 years old) patients with type 2 diabetes. The recruitment of patients is mainly selected from the patient database registered in Jiaozuo Second People's Hospital from 2016 to 2017, and the preliminary qualified patients are conducted by means of telephone interview or doctor's consultation recruitment.

(2) The subjects are in the charge of special personnel, so as to facilitate the management of related work, information recording and progress control.

(3) No matter what kind of recruitment method is adopted, the recruitment process is not mandatory.

(4) Relevant expenses involved in the recruitment process shall be explained clearly as far as possible, and general words shall be avoided to avoid misunderstanding.

(5) Pay special attention to avoid the following situations of recruiters

1. The patients who do not live in Jiaozuo cannot be guaranteed to participate in the training;

2. Subjects who are difficult to understand and comply with the program due to various reasons;

3. Subjects who are participating in other trial programs;

4. Subjects who tend to lead to disputes between doctors and patients.

# 18. General information collection of participants

The content of the collected participants' information includes: height, weight, gender, age, education, waist circumference, history of diabetes, medication and other diseases.

# 19. Baseline indicators and observation items

Baseline indicators: height, weight, waist circumference, medical history, medication history, BMI, heart rate, systolic blood pressure, diastolic blood pressure, blood sugar, glycated hemoglobin, C-peptide response, happiness index, emotional balance index, depression index, SF-36 scale.

Observation items: blood glucose, glycated hemoglobin, C-peptide response, happiness index, emotional balance index, depression index, sf-36 scale.

# 20. Standard operating procedures

Twelve weeks of training, five times a week, one hour of training each time, ten minutes of warm-up activities, ten minutes of relaxation activities, forty minutes of qigong, tai chi and stretching exercises of the same intensity. The qigong group chooses the qigong method developed and compiled by the qigong team of the school of physical education of zhengzhou university, which consists of 20 moves. Preparation type to moves 6 are preparation work, moves 7 to 15 are formal exercises, and moves 16 to 19 are finishing after practice. The whole set of exercises lasts 8-10 minutes. The tai chi group chooses the classic Chen style of tai chi, created by Chen zhenglei, which consists of 18 movements and lasts 5-6 minutes at a practice. The training of the stretching control group was mainly carried out by the professional fitness instructor with the same intensity and the body stretching exercise with the exercise equipment. Each action was repeated for 10-15 times according to its own endurance. It was mainly designed for the stretching and coordination of the body.

# 21. Statistical analysis method

The research group specially set up professionals of the laboratory department of jiaozuo second people's hospital to be responsible for the detection and collection of biochemical indicators (FPG, HbA1C and C-P) of type 2 diabetes patients. In addition, a data recording and collection analysis team was specially set up to conduct training on data recording and analysis during the experiment, mainly responsible for recording and analyzing the data of questionnaire survey of three groups of subjects before and after the intervention; Three coaches with more than 10 years of qigong, tai chi and fitness teaching experience are employed to conduct professional intervention practice teaching. During the teaching process, there are special personnel to supervise and count the students' practice, adverse reaction events and supervise the students' training at home. All the data are recorded and collected to establish a data archive, which is then analyzed by a special analyst.

The two-factor analysis of variance (ANOVA) method was adopted, and the test was set as diabetes (+/-) × the function method (+/-) to analyze the efficacy of the function method; Paired t test was used to analyze the parameter differences of participants before and after learning; Logistic regression was used to comprehensively analyze the efficacy, physiological effect and mental improvement effect of age, gender, function method and other factors on diabetes mellitus.

The statistical analysis will be calculated using SPSS 21.0 statistical analysis software. All statistical tests were conducted using the two-sided test method, and a p value less than or equal to 0.05 would be considered statistically significant.

# 22. Management system of participants

(1) Before the start of clinical trials, it is necessary to set up a core quality management team for the implementation of this project, and assign dedicated quality management personnel.

(2) Participants should formulate internal quality management strategies for the implementation of the project and clarify quality objectives.

(3) The research team

1. The research team should ensure that all participants are qualified to perform and complete the trial process.

2. Decompose tasks according to the test plan and clarify the division of responsibilities of each research team member.

3. A training mechanism was established within the research team to ensure that all participants had sufficient knowledge and information to complete the study.

4. As much as possible to ensure the stability of the research team.

# 23. Management system of specimen

This blood glucose test was completed at the Second People's Hospital of Jiaozuo, and all operating specifications and procedures met the requirements for specimen collection.

Collection: Strict implementation of aseptic technical procedures, disposable blood collection needles must be used, disposable blood collection needles after use must not be reused. One person, one needle and one piece (test paper) must be taken to avoid cross infection. Blood collection site usually USES the tip capillaries on both sides of the finger tip, edema, infection or callus of the site is not suitable for blood collection. Hang the arm for 10-15 seconds, wipe the blood collection area with a compound iodine medical cotton swab, and then conduct skin puncture after natural drying. After skin puncture, gently massage the finger to squeeze out the blood and place it on the designated area of the test paper. Press blood collection points with a dry cotton swab.

Submit for inspection: it shall be extracted by the inspection department and sent for inspection immediately after extraction.

Storage: Blood samples from the blood glucose test are tested immediately after collection. The blood samples collected from the blood glucose test are immediately tested after the blood glucose test. After the blood glucose test, they are kept by the hospital and sent to the medical waste treatment site for centralized treatment.

# 24. Management system of data

(1) Data collection

The patients carefully filled in the basic information questionnaire and the psychological questionnaire distributed by the members of the research team. The results of blood glucose test were given by Jiaozuo Second People's hospital.

(2) Data entry

Two data entry personnel input the data into the computer respectively.

(3) Data storage

The paper data is divided into groups and placed in a special archive bag in the school of physical education of Zhengzhou University (school-based Department). The electronic data is stored in a special hard disk, which is placed in the Physical Education College of Zhengzhou University (school headquarters).

# 25. Composition and responsibilities of data security and supervision committee

In order to ensure the smooth implementation of the project and minimize the risk of experimental research, the project team has set up a special data security and supervision committee. The team leader is Professor Si Hongyu, the host of the project, who is mainly responsible for overall supervision and data and safety work of experimental research. The deputy leader of data security is Professor Wang Zhenlong, who is mainly responsible for the design of the test and the safety and correctness of the data collection and recording process. Fan Haisheng is in charge of the Deputy group leader of the experiment, who is mainly responsible for the teaching supervision of qigong, tai chi and fitness and the standardization of action skills, so as to ensure the standardization, rationality and effectiveness of the intervention means. Other members include: special data collection and analysis personnel, recruitment and follow-up personnel, teaching supervision personnel, medical staff, professional laboratory personnel to strictly control the quality from all aspects. Ensure the safety, accuracy, specification and effectiveness of data in the whole test process.

# 26. Research team

| **Duties** | **Name** | **Division of labor** |
| --- | --- | --- |
| Principal | Si Hongyu | Organization and management |
|  | Wang Zhenlong | experimental design |
|  | Fan Haisheng | Provide skills |
| Main participants | Li Xiaoyuan | Data analysis |
|  | Chen Yamin | Data analysis |
|  | Zhang Yifeng | Experimental test |
|  | Tian Xinmao | Data acquisition |
|  | Li Huailiang | Teaching monitoring |
|  | Han Xiangyang | Teaching monitoring |
|  | Yin Ningning | Teaching monitoring |
|  | Yang Tianyu | Teaching monitoring |
|  | Zhang Qi | Recruiter |
|  | Xia Han | Recruiter |

# 27. Intellectual property

The research results of this project belong to the research team. The output related research papers, patents and other intellectual property rights belong to the research team of Zhengzhou University. In line with the principle of fairness and justice, the output of research results shall be based on the contribution of the work, and the order of the author's signature shall be negotiated by the partners. There is no relevant conflict of interest. If there is any objection in the negotiation, it shall be decided by the moderator of the project.

# 28. Publication plan

A research report will be completed by the time of project acceptance, and 1-3 papers are expected to be published.

# 29. Original data sharing plan

Not public

# 30. Treatment and management of participants after the trial

This study only examined the effect of exercise prescription on diabetes prevention and adjuvant therapy, as well as the improvement of physical quality, and compared the intervention effect of qigong and tai chi on the physical and mental health of diabetic patients. It's not about treatment.
